# Sarcopenic Obesity: Involvement of Oxidative Stress and Beneficial Role of Antioxidant Flavonoids

**DOI:** 10.3390/antiox12051063

**Published:** 2023-05-08

**Authors:** Un Ju Jung

**Affiliations:** Department of Food Science and Nutrition, Pukyong National University, 45 Yongso-ro, Nam-gu, Busan 48513, Republic of Korea; jungunju@naver.com or jungunju@pknu.ac.kr; Tel.: +82-51-629-5850

**Keywords:** sarcopenic obesity, pathophysiology, oxidative stress, flavonoids

## Abstract

Sarcopenic obesity, which refers to concurrent sarcopenia and obesity, is characterized by decreased muscle mass, strength, and performance along with abnormally excessive fat mass. Sarcopenic obesity has received considerable attention as a major health threat in older people. However, it has recently become a health problem in the general population. Sarcopenic obesity is a major risk factor for metabolic syndrome and other complications such as osteoarthritis, osteoporosis, liver disease, lung disease, renal disease, mental disease and functional disability. The pathogenesis of sarcopenic obesity is multifactorial and complicated, and it is caused by insulin resistance, inflammation, hormonal changes, decreased physical activity, poor diet and aging. Oxidative stress is a core mechanism underlying sarcopenic obesity. Some evidence indicates a protective role of antioxidant flavonoids in sarcopenic obesity, although the precise mechanisms remain unclear. This review summarizes the general characteristics and pathophysiology of sarcopenic obesity and focuses on the role of oxidative stress in sarcopenic obesity. The potential benefits of flavonoids in sarcopenic obesity have also been discussed.

## 1. Introduction

Obesity epidemics and aging are two major health concerns in developed countries. Over the last decade, obesity has become a worldwide pandemic [1]. Coupled with a rapidly aging population, obesity in the elderly is considered a major public health issue [2,3,4]. In general, obese elderly people have characteristics indicative of sarcopenia, such as loss of muscle mass, strength, and performance [5]. Although not clearly defined, the common definition of sarcopenic obesity is the coexistence of obesity and sarcopenia. The prevalence of sarcopenic obesity varies between 2% and 85% because of ambiguous criteria regarding its definition and diagnostic methods of sarcopenia and obesity [6,7,8,9]. Its prevalence is high among elderly people with comorbidities, such as type 2 diabetes, nonalcoholic fatty liver disease (NAFLD), dyslipidemia, and cardiovascular disease. Although the prevalence of sarcopenic obesity increases with age, younger generations are also at risk, especially when they have comorbidities [10,11]. 

Sarcopenic obesity can lead to various health problems such as metabolic syndrome, functional disability, osteoarthritis, osteoporosis, lung disease, renal disease, and depression [12,13,14,15]. Several mechanisms, including insulin resistance, inflammation, hormonal changes, behavioral problems, and oxidative stress, have been implicated in the pathophysiology of sarcopenic obesity [16,17,18,19]. To effectively prevent and treat sarcopenic obesity-related health problems, it is crucial to better understand sarcopenic obesity. 

Recently, there has been increasing interest in the therapeutic use of flavonoids for sarcopenic obesity. Flavonoids are bioactive molecules present in many plants including fruits, vegetables, grains, and spices. They provide a diverse range of health benefits including antioxidant, anti-inflammatory, anti-viral, anti-diabetic, anti-cancer, cardioprotective, and neuroprotective properties [20]. There is evidence showing that some flavonoids have the potential to prevent and treat sarcopenia and obesity via multiple mechanisms [21,22]. This article provides an overview of sarcopenic obesity, its underlying pathophysiological mechanisms, and the potential benefits of flavonoids for the prevention and treatment of sarcopenic obesity. 

## 2. General Characteristics

A rational consideration of sarcopenia and obesity is required to diagnose and treat sarcopenic obesity. 

### 2.1. Sarcopenia

Sarcopenia is characterized by the loss of skeletal muscle mass, strength, and physical function, which can lead to physical disability, lower quality of life, and a higher mortality rate [23,24,25]. Although age-related changes in skeletal muscle are the primary causes of sarcopenia, they can also occur due to various factors, such as limited mobility, malnutrition, and obesity [26,27]. According to the definition provided by the European Working Group on Sarcopenia in Older People (EWGSOP), the diagnostic criteria for sarcopenia include low muscle strength (an early indicator of sarcopenia), low muscle mass, and low physical performance [26]. Specifically, subjects with low muscle strength may have sarcopenia, and low muscle quantity or quality should be confirmed before diagnosis. Subjects who fulfil all three criteria are considered to have a severe sarcopenia. Various tests and tools are used to diagnose sarcopenia in clinical practice and research. Grip strength and chair stand tests are two commonly used tests of skeletal muscle strength. Muscle quantity can be determined using various tools, including magnetic resonance imaging, computed tomography, dual-energy X-ray absorptiometry, and bioelectrical impedance analysis. Gait speed is commonly used for the assessment of physical performance, and a short physical performance battery and the timed up-and-go test are alternatives to the gait speed test. Recently, the importance of muscle strength (assessed by grip strength) and physical performance (assessed by gait speed) over muscle mass in the definition of sarcopenia is being emphasized [28].

Sarcopenia is a known risk factor for various health issues, including chronic disease progression, physical disability, falls, osteoporosis, and postoperative complications (both infections and noninfectious complications) [29,30,31,32,33,34]. Therefore, it is associated with increased hospital costs, length of hospital stay, and recurrence [35]. Although early diagnosis is key to preventing and treating sarcopenia-related health problems, there is no global consensus on the definition and diagnostic criteria for sarcopenia. 

### 2.2. Obesity

Obesity is defined as abnormal or excessive fat accumulation that may be a health hazard [36]. According to the World Health Organization, a body mass index (BMI) ≥ 30 kg/m^2^ in adults indicates obesity. However, it is recommended to use a lower BMI cut-off point for defining obesity (≥25 kg/m^2^) in the Asian population, due to a higher risk of obesity-related diseases such as diabetes [37]. Waist circumference, another index of obesity, is the most frequently used to measure abdominal adiposity. Abdominal obesity is defined as a waist circumference ≥88 cm and ≥102 cm for women and men, respectively [38]. Waist circumference criteria for diagnosis of abdominal obesity also differ depending on ethnicity. It occurs when energy intake exceeds energy expenditure, which is influenced by genetic and environmental factors (e.g., nutrition, exercise, viruses, microbiome, and circadian rhythms). Obesity is an important risk factor for metabolic abnormalities, including insulin resistance, type 2 diabetes, dyslipidemia, NAFLD, and cardiovascular diseases. It is also associated with cancer, osteoarthritis, pulmonary dysfunction (e.g., obstructive sleep apnea), and cognitive impairment [39,40,41]. Recently, obesity has been reported to be a risk factor for severe coronavirus disease 2019 (COVID-19) [42]. 

### 2.3. Sarcopenic Obesity

The obesity epidemic is increasing worldwide [43]. The obesity epidemic is prevalent not only in industrialized but also in developing countries [43]. In addition to the prevalence of obesity in the general population, the incidence of obesity in the elderly has been increasing and poses a serious threat to the health of elderly individuals in both developed and developing countries [44]. It is particularly noteworthy that the prevalence of sarcopenic obesity is increasing with the rising population of senior citizens and has become an important health issue. Recently, sarcopenic obesity has been considered a health risk in younger and older populations [10,11].

Sarcopenia commonly occurs with aging and is often accompanied a relative or absolute increase in adiposity. However, it can strike anyone with obesity, regardless of age. Obesity can independently cause sarcopenia, because underlying factors contributing to obesity such as inflammation, insulin resistance, and oxidative stress can adversely affect sarcopenia [45]. Meanwhile, sarcopenia can promote fat accumulation due to decreased physical activity and energy expenditure. Thus, obesity and sarcopenia may affect each other. Although the definition of sarcopenic obesity remains under discussion, most studies have defined it based on the concomitant presence of sarcopenia and obesity. For example, based on a recent consensus statement by the European Society for Clinical Nutrition and Metabolism (ESPEN) and the European Association for the Study of Obesity (EASO), sarcopenic obesity is defined as abnormal and excessive adiposity and low skeletal muscle mass and function [46]. The ESPEN and EASO recommended following diagnostic criteria. Screening for sarcopenic obesity is performed to verify the presence of both high BMI or waist circumference (based on ethnic cut-off points) and surrogate indicators of sarcopenia (clinical symptoms, clinical suspicion, or questionnaires). After positive screening results, both altered skeletal muscle functional parameters (e.g., hand grip strength) and altered body composition (increase in % fat mass and decrease in muscle mass) are required to make a firm diagnosis of sarcopenic obesity. Once a diagnosis of sarcopenic obesity is confirmed, it can be classified into two stages depending on the presence or absence of complications (e.g., metabolic diseases, functional disabilities, and cardiovascular and respiratory diseases). Since an internationally agreed definition and diagnostic criteria for sarcopenic obesity have not been established, further efforts are needed to reach a consensus on an appropriate definition and diagnostic criteria. 

## 3. Pathophysiological Mechanisms: Involvement of Oxidative Stress 

**Figure 1:**
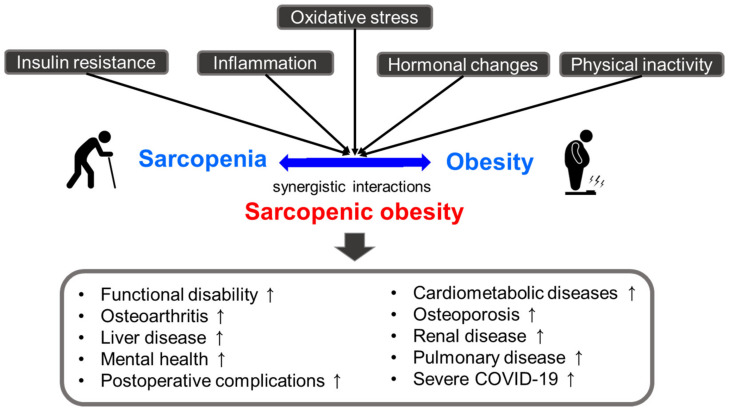
Pathophysiologic characteristics and consequence of sarcopenic obesity.

Obesity and sarcopenia have common pathophysiological characteristics, including insulin resistance, inflammation (increased secretion of pro-inflammatory markers and decreased anti-inflammatory markers), hormonal changes (a decline in growth hormone, testosterone, and estrogen), lack of physical activity, and oxidative stress [16,17,18,19]. As a result of their synergistic interactions, sarcopenic obesity may have deleterious effects on health compared with those of either condition alone [47,48,49,50,51,52,53,54,55,56,57]. Several studies have suggested that sarcopenic obesity is linked to an increased risk of functional disability, cardiometabolic diseases (cardiovascular disease, type 2 diabetes, and hypertension, among others), and other comorbidities, such as liver disease, osteoarthritis, osteoporosis, pulmonary disease, renal disease, mental health problems (cognitive impairment and depression, among others), postoperative complications, and severe COVID-19 [47,48,49,50,51,52,53,54,55,56,57] (Figure 1). 

Oxidative stress is a critical factor that links sarcopenic obesity with related comorbidities [17]. It is also associated with other factors contributing to sarcopenic obesity, such as insulin resistance, inflammation, hormonal changes, and behavioral problems [17]. Oxidative stress is caused by an imbalance between the formation of free radicals (highly reactive molecules containing one or more unpaired electrons) and antioxidant defenses, which can damage many tissues and lead to various diseases, including sarcopenic obesity [58]. Free radicals, such as reactive oxygen species (ROS) and reactive nitrogen species (RNS), are derived from endogenous and exogenous sources. Endogenous antioxidants (e.g., superoxide dismutase, catalase, glutathione peroxidase, and bilirubin) and exogenous antioxidants (e.g., ascorbic acid, β-carotene, α-tocopherol, and phenolic compounds) can defend against free radical-induced damage [58]. However, insufficient antioxidant capacity and increased ROS and RNS generation can cause oxidative damage to organelles, carbohydrates, proteins, nucleic acids, and lipids, leading to dysfunction and disease development [59]. In elderly individuals, oxidative stress elicits sarcopenia and obesity through mitochondrial dysfunction, endoplasmic reticulum (ER) stress, and imbalances in muscle mass control [17]. In this section, we focused on the effects of oxidative stress on the development of sarcopenic obesity.

### 3.1. Mitochondrial Dysfunction

Mitochondrial dysfunction can increase ROS production [60] because mitochondria are important sites for ROS formation [61]. The release of ROS from mitochondria increases with aging, resulting in increased oxidative damage to mitochondrial and cellular proteins, lipids, and DNA, which consequently causes a decline in mitochondrial function, including mitochondrial protein synthesis, respiration, and maximal ATP production rate [62,63,64,65]. 

The removal of dysfunctional mitochondria via mitophagy (a selective form of autophagy) is critical for maintaining the redox balance and muscle health. A lack of capacity of muscles to effectively remove dysfunctional mitochondria can contribute to excessive ROS formation and a consequent decrease in mitochondrial quantity and quality, which can lead to the development of muscle fiber atrophy in sarcopenia [66]. Muscle atrophy resulting from aging can occur due to a decrease in the total number of muscle fibers and a simultaneous decrease in the size of individual fibers [67,68]. Aging-related muscle loss was mainly attributed to a decrease in type II muscle fibers, probably because type II fibers embedded with less mitochondria are more vulnerable to aging-induced processes [69,70].

Oxidative stress associated with obesity can also trigger disturbances in mitochondrial function, resulting in increased ROS production. Although white adipose tissue (WAT) is not a mitochondria-rich tissue, mitochondria in WAT have a critical role in maintaining metabolic homeostasis, including adipogenesis, lipogenesis, lipolysis, and adipokine production [71,72]. Obesity caused by excess energy intake, such as HFD, could lead to mitochondrial dysfunction in adipocyte partly due to ROS-induced oxidative damage to the adipocyte. Consequently, mitochondrial dysfunction caused by oxidative stress in obesity can induce insulin resistance, inflammation, dyslipidemia, and other adverse effects [71,72]. Although the mechanism underlying adipocyte mitochondrial dysfunction in obesity is unclear, hypertrophic adipocytes (enlargement of adipocyte size) in obesity is associated with mitochondrial dysfunction [73]. Another possibility is that a rise in mitochondrial substrate overload in conditions of overnutrition increases electron transport chain activity in the mitochondria and subsequently ROS generation, leading to oxidative stress [74]. Nutrient overload in obesity can also lead to ER stress, which promotes oxidative stress and contributes to mitochondrial dysfunction in adipocyte [75]. 

The protein kinase A (PKA)/liver kinase B1 (LKB1)/AMP-activated protein kinase (AMPK) signaling pathway plays essential roles in improving mitochondrial dysfunction and inhibiting oxidative stress [76,77]. PKA activates AMPK, a crucial cellular energy sensor, via LKB1, which promotes mitochondrial biogenesis and antioxidant capacity in the skeletal muscles [76,77].

### 3.2. ER Stress

The ER performs many functions, particularly in protein synthesis and folding. The excessive formation of unfolded or misfolded proteins in the ER can lead to ER stress [78]. As redox homeostasis is crucial for protein folding in the ER, oxidative stress can generate misfolded proteins by disrupting protein folding and thus elicits ER stress. ER stress is a major source of ROS, which accelerates oxidative stress. ER stress affects cellular homeostasis and morphology, leading to various diseases, including obesity and sarcopenia. 

ER stress is commonly observed in obese subjects and in genetically modified or high-energy diet-induced obese animals [79,80,81]. Chronic ER stress in the liver, skeletal muscle, and adipose tissue impairs insulin sensitivity and contributes to inflammation, leptin resistance, and steatosis [79,80,81,82,83]. By contrast, weight loss in obese individuals decreases ER stress and increases insulin sensitivity [84]. Moreover, a reduction in ER stress in the adipose tissue and liver using chemical or molecular chaperones was found to improve inflammation, hepatic steatosis, and glucose homeostasis in obese mice [85,86], suggesting that prolonged ER stress may be involved in obesity-associated tissue dysfunction and metabolic disturbances. 

ER stress is also an important contributor to sarcopenia development. Skeletal muscle contains the ER, which plays a critical role in the regulation of calcium storage and protein homeostasis. ER stress is increased in the skeletal muscles of both aged people and rodents [87,88,89,90], and oxidative stress is involved in the induction of ER stress in obese subjects with and without comorbidities [91]. Increased ER stress was found to cause diaphragm contractile dysfunction in a muscle atrophy mouse model due to sepsis [92]. In contrast, the inhibition of ER stress protected against muscle atrophy in a hind-limb-unloaded mouse model, which showed skeletal muscle atrophy and weakness [93]. 

### 3.3. Imbalance in Muscle Mass Control

The balance between the anabolic and catabolic pathways is important for maintaining muscle mass. Oxidative stress causes muscle wasting by activating the catabolic pathways and inhibiting the anabolic pathway [59]. 

#### 3.3.1. Anabolic Pathway

In skeletal muscle, phosphatidylinositol 3-kinase (PI3K)/protein kinase B (Akt)/mammalian target of rapamycin (mTOR) pathway is one of the major anabolic pathways controlling protein synthesis [94,95]. The activation of PI3K phosphorylates and activates AKT, which regulates several downstream molecules, including mTOR [96]. mTOR is a key regulator of protein synthesis in addition to having a role in other biological functions, such as the regulation of cell growth and survival. It regulates the anabolic and catabolic signaling pathways in skeletal muscles and thus modulates muscle hypertrophy and muscle wasting [97]. According to the free radical theory of aging [98], ROS damage mitochondrial proteins and reduce ATP generation, resulting in the inhibition of the mTOR pathway and subsequent down-regulation of protein synthesis. Furthermore, the impairment of the mTOR pathway has been observed in patients with sarcopenia [99]. Other studies also support the deleterious effect of mTOR inhibitors on muscle mass and growth [100,101]. 

The PI3K/Akt/mTOR pathway is activated by several upstream triggers, such as insulin-like growth factor 1 (IGF-1), insulin, and physical activity, all of which are associated with obesity or aging [95,102]. Circulating IGF-1 levels are lower in sarcopenic subjects than in non-sarcopenic subjects, and its levels are associated with sarcopenia in the elderly as well as fat mass and the prevalence of comorbidities in obese subjects [103,104,105]. Moreover, insulin and IGF-1 stimulate protein synthesis in the skeletal muscles of mice [106], and impaired insulin activity in skeletal muscles is linked to obesity and sarcopenia [107]. Type 2 diabetic db/db mice show an impaired PI3K/Akt pathway along with increased protein degradation, resulting in muscle atrophy, whereas an improvement in insulin-sensitivity using rosiglitazone protects against muscle loss [108,109]. Physical activity is most effective in preventing sarcopenia in elderly individuals [109]. Exercise can mitigate a decline in autophagy in skeletal muscles of aged rats by regulating the mTOR pathway, resulting in improved aging-induced skeletal muscle atrophy [110]. However, the role of the mTOR pathway in sarcopenia remains controversial [111]. Aging is influenced by mTOR hyperactivation [112].

#### 3.3.2. Catabolic Pathway

The loss of skeletal muscle occurs in a state of protein degradation that exceeds its synthesis. In skeletal muscle, protein degradation is mainly mediated by two catabolic pathways: the ubiquitin–proteasome pathway and the autophagic/lysosomal pathway. The ubiquitin–proteasome pathway is crucial for protein degradation in striped muscles [113]. FoxO activates the expression of the genes involved in the ubiquitin-proteasome pathway, including atrogin-1, muscle atrophy F-box (MAFbx), and muscle RING-finger protein-1 (MuRF1), which are up-regulated in muscle atrophy [114,115]. FoxO also regulates the activation of autophagic/lysosomal pathway-related genes (LC3, Bnip3, and Bnip3l) during muscle atrophy in vivo [116]. The activation of Akt not only induces mTOR signaling but also inhibits FoxO through phosphorylation, thereby inhibiting the catabolic process of skeletal muscle [117]. Autophagy is a catabolic pathway that removes dysfunctional organelles and denatured proteins in a lysosome-dependent process [118]. With aging, abnormal autophagy causes the accumulation of damaged cellular constituents, such as damaged mitochondria, which increases ROS generation and further damage [119]. Dysregulated autophagy in muscles also causes ER stress, impaired sarcomeric protein turnover, and cell death, resulting in the loss of muscle mass [120]. Both a lack of and excessive autophagy are associated with muscle atrophy [119,120]. 

Multiple triggers including oxidative stress and inflammatory cytokines affect protein degradation. Oxidative stress-induced atrophy is associated with the up-regulation of FoxO1 and MuRF1 expression in muscle cells, whereas treatment with ascorbic acid, an antioxidant, counteracts oxidative stress-induced atrophy by down-regulating FoxO1, MuRF1, and atrogin-1 expression [121]. ROS also activates autophagy through multiple mechanisms, leading to protein breakdown [122]. Another important factor controlling protein degradation and skeletal muscle loss is nuclear factor-kappa B (NF-κB). The activation of NF-κB results in skeletal muscle atrophy by increasing the expression of ubiquitin–proteasome pathway protein (e.g., MuRF1), pro-inflammatory cytokines (e.g., TNF-α and IL-1), and chemokines contributing to muscle loss [123]. Increased levels of NF-κB and pro-inflammatory cytokines are observed in elderly people with sarcopenia, as well as in obese individuals [124,125,126,127]. 

#### 3.3.3. Satellite Cells

Satellite cells, also known as muscle stem cells, are the primary source for muscle re-generation. It is critical for muscle fiber maintenance, repair, and remodeling [128]. Age-related reductions in the number and function of satellite cells occur, especially in type II fibers, although conflicting results have been reported [129,130,131,132]. In addition, patients with sarcopenia frequently exhibit aberrant satellite cell homeostasis [133]. Although its role in the development of sarcopenia and sarcopenic obesity is not completely clear, exercise-induced satellite cell activation, along with the provision of sufficient nourishment, appears to offer effective protection against sarcopenia and sarcopenic obesity [134]. 

Considerable evidence suggests that excess ROS and diminished antioxidant capacity can impair muscle regeneration, primarily by affecting satellite cell homeostasis [135,136]. An increase in ROS occurs in the satellite cells of aged muscles, and antioxidant capacity is decreased in aged satellite cells [136,137]. Oxidative stress not only leads to impaired removal of misfolded proteins but also dysregulates basal autophagy, which is vital for the maintenance of the stem cell quiescent state, contributing to stem cell resilience [138], thereby affecting muscle regeneration [135,136,139,140]. In addition, redox-sensitive signaling pathways, such as Notch, Wnt, p38/MAPK, and JAK/STAT3, are aberrantly expressed in satellite cells during aging, causing abnormal satellite cell functions, including proliferation, fibrosis, and differentiation [141,142,143,144]. 

Myogenic regulatory factors (e.g., Myf5, MyoD, Myogenin, and MRF4) play a critical role in controlling the myogenesis via satellite cells. The expression of myogenic regulatory factors is up-regulated during myogenesis and influences the activation and differentiation of stem cells [145]. By contrast, myostatin, a myokine secreted by myocytes, functions as a negative regulator of muscle growth and regeneration by reducing satellite cell number and regeneration, inhibiting the Akt/mTOR pathway, and activating FoxO [146]. Although the role of myostatin in aging and other muscle-wasting conditions is unclear, skeletal muscle myostatin mRNA expression is higher in overweight and obese middle-aged and older adults with sarcopenia than in those without sarcopenia, and myostatin mRNA expression is positively correlated with BMI, fat mass, and mid-thigh intra-muscular fat area [147]. Genetic and pharmacological inhibition of myostatin ameliorates sarcopenic obesity by increasing muscle mass and improving glucose homeostasis [147,148,149,150,151,152]. 

## 4. Effects of Antioxidant Flavonoids on Sarcopenic Obesity

**Figure 2:**
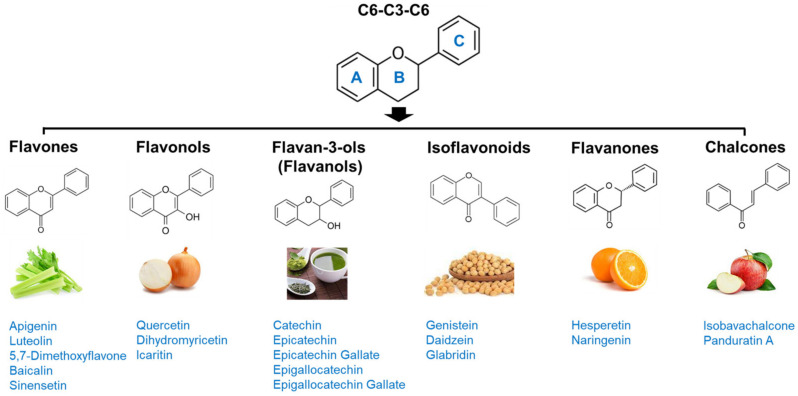
Subgroups of flavonoids and their chemical structures.

Flavonoids are among the most abundant phenolic compounds in edible plants [153]. More than 5000 flavonoids have been reported to date [154]. They have a C6-C3-C6 backbone and are generally subdivided into different subgroups based on their chemical structures: flavones, flavonols, flavan-3-ols, chalcones, flavanones, and isoflavonoids (Figure 2). Flavonoids possess many bioactivities, such as antioxidant, anti-inflammatory, and anti-viral effects, and protect against various diseases, including cardiovascular diseases, diabetes, neurodegenerative diseases, osteoporosis, and cancer [20]. In addition, in vitro and in vivo studies have reported their anti-obesity potential via stimulation of energy expenditure, appetite suppression, inhibition of adipocyte differentiation and digestive enzymes, promotion of adipocyte apoptosis and lipolysis, and regulation of lipid metabolism and gut microbiota [21]. Recently, flavonoids have received renewed attention as candidates for improving muscle atrophy and muscle health [22]. Some flavonoids have been reported to provide effective protection against sarcopenic obesity; therefore, their anti-sarcopenic effects and mechanisms are summarized.

### 4.1. Apigenin

Apigenin (4′,5,7-trihydroxyflavone) is a flavone that is plentiful in edible plants such as parsley, celery, celeriac, oranges, and chamomile [155]. There is evidence that it has positive health effects, owing to its anti-inflammatory, antioxidant, anti-steatotic, anti-diabetic, anti-cancer, and neuroprotective properties [156,157,158,159,160,161]. Apigenin also possesses anti-obesity properties associated with decreased food intake, increased energy expenditure, activated lipolysis, fatty acid oxidation, and control of gut microbiome composition [162,163,164]. 

In addition, apigenin exerts a protective effect against sarcopenia. In lipopolysaccharide (LPS)-treated mouse skeletal muscle cells, it decreases the expression of the atrophic genes atrogin-1 and MAFbx [165]. This anti-atrophic effect was supported by the findings of an in vivo study in which apigenin increased muscle mass and enhanced muscle function in mice by enhancing skeletal muscle hypertrophy and myoblast differentiation [166]. Apigenin also alleviates sciatic nerve denervation-induced muscle atrophy by inhibiting muscle inflammation [167]. Moreover, apigenin not only decreases fat mass but also prevents muscle loss and increases exercise capacity by down-regulating atrogin-1 and MuRF1 in the skeletal muscle of high-fat diet (HFD)-induced obese mice [168], which suggests that it can ameliorate HFD-induced sarcopenic obesity. Interestingly, a recent study reported that apigenin improves muscle atrophy by decreasing oxidative stress and activating mitophagy and apoptosis in aged mice [169]. 

### 4.2. Luteolin 

Another flavone luteolin (3′,4′,5′,7′-tetrahydroxyflavone), commonly occurring in edible plants such as celery, parsley, and broccoli [170], has a protective effect on sarcopenic obesity [171]. Long-term supplementation with luteolin not only decreases body weight and fat mass but also increases muscle mass, muscle fiber size and number, and muscle function in HFD-induced obese mice. The beneficial effects of luteolin on sarcopenic obesity are associated with suppressed protein degradation, decreased muscular lipid accumulation, and attenuated inflammation. The anti-atrophic properties of luteolin are supported by other in vitro and in vivo studies using LPS- and cachexia-induced muscle atrophy models [165,172]. 

### 4.3. Quercetin

Quercetin (3,3′,4′,5,7-pentahydroxyflavone) is a flavonol abundantly present in various vegetables and fruits [173]. It can protect against multiple diseases, such as osteoporosis, cancer, memory impairment, and cardiovascular diseases, and these promising effects are achieved partly through its antioxidant and anti-inflammatory actions [174,175,176,177]. Quercetin also has a positive influence on decreasing body weight and adiposity in diet-induced obese animals, and multiple mechanisms, such as the activation of lipolysis, inhibition of lipogenesis and adipogenesis, and browning effects, have been implicated in its anti-obesity effects in vivo and in vitro [178,179,180,181,182,183,184]. In particular, quercetin inhibits oxidative stress and NF-κB and thus limits immune activation and inflammation, which results in an improvement in mitochondrial functions in the adipose tissue of HFD-induced obese mice [185]. Clinical trials have supported the anti-obesity effects of quercetin. Quercetin administration reduces BMI and fat mass in overweight or obese individuals [186]. In another study conducted on overweight/obese subjects with various apolipoprotein E genotypes, reductions in waist circumference and triglyceride levels were observed after quercetin administration [187]. However, quercetin slightly increases pro-inflammatory TNF-α levels [187], and high doses of quercetin do not affect oxidative stress in obese people [188]. Further clinical studies are required to investigate its anti-obesity effects.

The protective effects of quercetin on obesity-induced skeletal muscle atrophy have also been demonstrated in vivo [189,190]. In the skeletal muscle of HFD-fed mice, quercetin was found to reverse the increase in levels of mRNA and protein of atrophic markers (atrogin-1, MuRF1) and pro-inflammatory markers (TNF-α, MCP-1) induced by HFD [189]. The anti-atrophic effect of quercetin is reported in another study showing that it suppresses muscle atrophy in HFD-induced obese mice via up-regulation of Nrf2-mediated heme oxygenase-1 and antioxidant capacity and down-regulation of pro-inflammatory NF-κB [190]. Therefore, quercetin may protect against obesity-induced sarcopenia and metabolic dysregulation.

Its anti-atrophic effects have been observed in other animal models [181,182,183,184]. Quercetin increases muscle mass and suppresses unloading-induced disused muscle atrophy and lipid oxidation in mice [191]. Mukai et al. [192] have also found that quercetin inhibits denervation-induced muscle loss by increasing p-Akt, IGF-1, and PGC-1α in mice, despite having no effect on MuRF1. A recent study demonstrated the protective role of quercetin in cancer- and chemotherapy-induced muscle loss by maintaining mitochondrial homeostasis [193]. It is particularly noteworthy that quercetin promotes behavioral functional recovery by hastening the recovery of weight in damaged muscles and increasing neuronal intrinsic growth capacity following sciatic nerve crush injury in mice [194]. In addition, in middle-aged and older adults, the administration of quercetin glycoside together with low-intensity resistance exercise improves muscle quality (an increase in muscle stiffness) and independent muscle quantity [195].

### 4.4. Dihydromyricetin

Dihydromyricetin, also known as ampelopsin, is a flavonol present in medicinal herbs, vegetables, and fruits, including grapes and berries [196]. It has multiple biological and pharmacological properties, including antioxidant, anti-inflammatory, anti-cancer, neuroprotective, and hepatoprotective actions [197,198,199,200,201]. Some studies have also demonstrated that dihydromyricetin prevents obesity in HFD-fed and ob/ob mice and that its role in adipogenesis inhibition, adipocyte browning activation, and gut microbiota regulation is related to its anti-obesity effect [202,203,204,205]. 

In addition, Zhou et al. [206] reported that dihydromyricetin improved skeletal muscle insulin resistance and reduced the proportion of type I fibers in HFD-induced obese and ob/ob mice. Type I fibers have more mitochondria and mostly involve fatty acid oxidative phosphorylation for energy generation compared to type II fibers [207]. Zou et al. [208] demonstrated that dihydromyricetin improved hypobaric hypoxia-induced mitochondrial dysfunction in rats, resulting in improved physical performance. The protective effects of dihydromyricetin on mitochondrial function and muscle atrophy are supported by another study, which showed that it ameliorates dexamethasone-induced muscle atrophy by improving mitochondrial dysfunction [209]. Accordingly, it down-regulated FoxO3a-mediated protein degradation and up-regulated Akt/mTOR pathway-dependent protein synthesis. Huang et al. [209] also observed that dihydromyricetin suppressed dexamethasone-induced oxidative stress in skeletal muscles, which might contribute to protection against muscle atrophy because oxidative stress causes muscle wasting by activating the catabolic pathway and inhibiting the anabolic pathway [59]. Beneficial effects of dihydromyricetin on muscle atrophy induced by inflammation or D-galactose (a useful agent for accelerating aging) have also been reported in vitro and in vivo [210,211]. 

### 4.5. Epigallocatechin Gallate and Epicatechin

Epigallocatechin gallate (EGCG) is a flavanol abundantly present in tea, especially green tea, and is also present in small amounts in other plant foods, such as apples, carobs, berries, and avocados [212,213]. It possesses diverse bioactivities and pharmacological effects, including antioxidant, anti-inflammatory, cardioprotective, anti-diabetic, and anti-cancer effects [214,215,216,217,218]. EGCG also has positive effects on weight loss and fat reduction in HFD-induced obese animals [219,220,221,222,223]. The anti-obesity effect is linked to inhibited adipogenesis and lipogenesis, increased lipolysis, brown fat thermogenesis, mitochondria biogenesis, white fat autophagy, and improved gut microbiota homeostasis [219,220,221,222,223,224,225]. However, its potential benefits in obese individuals remain obscure [226,227,228,229].

The existing evidence indicates the beneficial effect of EGCG in alleviating sarcopenia. In aged rats, EGCG increases the recovery of muscle mass and function after disuse by de-creasing apoptotic signaling and increasing satellite proliferation [230]. Takahashi et al. [231] reported that EGCG up-regulates autophagy signaling to improve the clearance of damaged organelles in resting and unloaded conditions but selectively inhibits autophagy-related proteins (Beclin1 and LC3) in reloaded muscles of aged rats, perhaps leading to the recovery of muscle mass and function. In another study using aged animals, EGCG prevented muscle loss by inhibiting protein degradation via the ubiquitin–proteasome pathway (atrogin-1, MuRF1, myostatin), along with the up-regulation of the anabolic factor IGF-1 [232,233]. In line with these findings, EGCG prevents cachexia-induced muscle loss via down-regulation of NF-κB, atrogin-1, and MuRF1 in mice [234], and it recovers muscle function impairment and damaged muscle fibers following nerve injury in rats by activating the anti-apoptotic signaling pathway [235]. Moreover, EGCG enhances endurance capacity in mice [236], decreases oxidative stress, and alleviates mitochondrial dysfunction by decreasing excessive autophagy in the skeletal muscles of rats with type 2 diabetes [237]. The anti-sarcopenic effect of EGCG is associated with not only decreased protein degradation but also increased protein synthesis in skeletal muscle [238]. 

Epicatechin, another flavanol found in green tea, also hinders aging-associated skeletal muscle degeneration and enhances physical activity in mice [239]. Similarly, supplementation of epicatechin in obese middle-aged mice (16 months of age) not only decreases fat mass but also enhances physical performance by increasing muscle growth and differentiation and by decreasing the ubiquitin–proteasome degradation pathway [240]. Interestingly, epicatechin reversed aging-induced oxidative stress and mitochondrial biogenesis in vivo [241]. In vitro studies also support its anti-atrophic effects via inhibition of protein degradation and improvements in mitochondrial biogenesis and muscle growth in skeletal muscle cells [242,243]. In addition, the administration of epicatechin together with resistance training increases skeletal muscle strength and growth factors in elderly individuals with sarcopenia [244]. The combination of tea catechins and exercise has a positive effect on maintaining muscle mass and physical function in the elderly compared to those without catechins [245]. However, there was no association between green tea consumption and sarcopenia in menopausal women [246]. Moreover, few studies have assessed the effects of EGCG on sarcopenic obesity in humans.

### 4.6. Others

5,7-dimethoxyflavone, a flavone found in Kaempferia parviflora, also exerts protective effects on sarcopenia and obesity [247,248]. Supplementation of 5,7-dimethoxyflavone to aged mice improves skeletal muscle function and increases muscle mass by increasing mitochondrial biogenesis, activating protein synthesis via the mTOR pathway, and suppressing protein degradation via the FoxO pathway [247]. 5,7-dimethoxyflavone prevents obesity by suppressing adipogenesis in HFD-fed obese mice [248].

Glabridin, an isoflavan found in licorice, prevents glucocorticoid-induced muscle atrophy in vivo and in vitro [249]. Furthermore, it was found to ameliorate HFD-induced obesity by inhibiting lipid synthesis in the WAT and increasing muscular fatty acid oxidation via regulation of mitochondrial function as AMPK activator [250]. 

Hesperetin, a major flavanone abundant in citrus fruits, such as lemons and oranges, decreases body weight and fat mass in HFD-induced obese mice [251]. It also increases muscle fiber size and enhances running performance in aged mice [252]. These beneficial effects are associated with activation of PGC-1α and Nrf2 along with increased antioxidant capacity. Recently, Yeh et al. [253] reported that long-term oral administration of hesperetin improves age-associated energy expenditure decline, fat accumulation, and muscle loss in aged mice. 

Another citrus flavanone, naringenin, reportedly protects against HFD-induced adiposity and inflammation in ovariectomized mice [254]. Moreover, it increases muscle mass and locomotor activity and decreases muscle lipid accumulation. Similarly, in ovariectomized mice fed a normal diet, naringenin improved estrogen deficiency-induced fat accumulation and muscle loss [255]. Previous studies suggest that naringenin increases energy expenditure, improves insulin resistance, and regulates skeletal muscle differentiation by controlling estrogen receptor α and β signal pathway [256,257]. 

Other flavonoids, such as daidzein (isoflavone), genistein (isoflavone), baicalin (fla-vone), sinensetin (flavone), icaritin (flavonol), isobavachalcone (chalcone), and panduratin A (chalcone), have the potential to attenuate obesity and sarcopenia [258,259,260,261,262,263,264,265,266,267,268,269,270,271,272]. 

## 5. Conclusions

**Figure 3:**
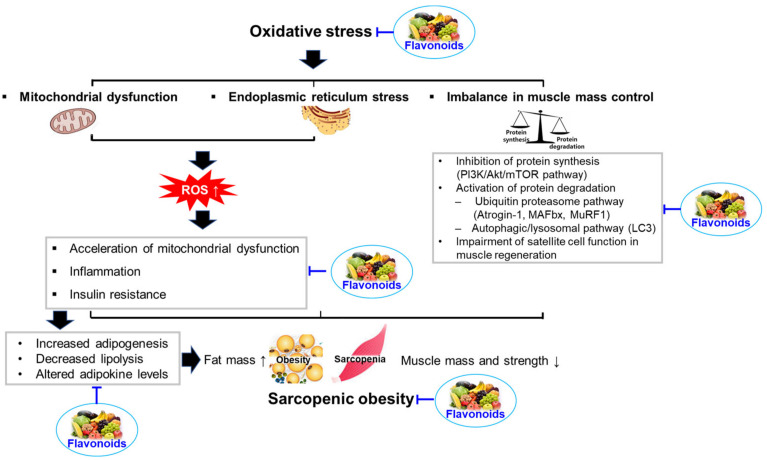
Summary of the influences of oxidative stress in the development of sarcopenic obesity and proposed mechanism by which flavonoids can protect against sarcopenic obesity.

Sarcopenic obesity has become a global epidemic affecting all generations, including the elderly. In addition to age-related diseases, multiorgan dysfunction and multiple mechanisms are implicated in the development of sarcopenic obesity. The development and progression of sarcopenic obesity are related to oxidative stress, inflammation, insulin resistance, hormonal changes, and behavioral problems. Accumulating evidence suggests that flavonoids may be effective in preventing and treating sarcopenic obesity, owing to their ability to control oxidative stress, inflammation, insulin resistance, mitochondrial dysfunction, anabolic and catabolic pathways, and satellite cells (Figure 3). Flavonoids having potential for improving sarcopenic obesity are summarized in Table 1.

However, the underlying mechanisms of action remain obscure, and limited human clinical trials have investigated the effectiveness of flavonoids in sarcopenic obesity. Therefore, further studies are required to assess the potential impact and mechanism of flavonoid-mediated prevention of sarcopenic obesity. In addition, advancing the understanding of sarcopenic obesity may contribute to the emergence of novel therapies to prevent associated comorbidities, such as cardiometabolic diseases.

## Figures and Tables

**Table 1:** Summary of flavonoids having potential for improving sarcopenic obesity.

Subclass	Flavonoid Name	Experimental Model	Effects and Potential Mechanisms	References
Flavone	Apigenin	HFD-fed C57BL/6J mice	Body weight gain ↓, Food intake ↓	[162]
		HFD-fed C57BL/6J mice	Body weight gain ↓, Energy expenditure ↑, lipolysis ↑, fatty acid oxidation ↑	[163]
		HFD-fed C57BL/6J mice	Body weight ↓, gut microbiota regulation	[164]
		LPS-treated C2C12 myotubes	Protein degradation (atrogin-1, MAFbx) ↓	[165]
		C57BL/6J mice	Muscle mass ↑, muscle function ↑	[166]
		C2C12 myotubes	Stimulation of myogenic differentiation	[166]
		Denervated mice	Muscle mass ↑, protein degradation (MuRF1) ↓, Muscle inflammation ↓	[167]
		HFD-fed C57BL/6J mice	Fat mass ↓, muscle mass ↑, protein degradation (atrogin-1, MuRF1) ↓, mitochondrial dysfunction ↓, mitochondrial biogenesis ↑, AMPK ↑	[168]
		PA-treated C2C12 myotubes	Muscle atrophy ↓, mitochondrial dysfunction ↓, AMPK ↑	[168]
		Aged mice	Muscle mass ↑, muscle function ↑, mitochondrial function ↑, mitochondrial biogenesis ↑, oxidative stress ↓, hyperactive mitophagy and apoptosis ↓	[169]
Flavone	Luteolin	LPS-treated C2C12 myotubes	Protein degradation (atrogin-1, MAFbx) ↓	[165]
		HFD-fed C57BL/6J mice	Body weight ↓, fat mass ↓, muscle mass ↑, muscle fiber size and number ↑, muscle function ↑, protein degradation (atrogin, FoxO, MuRF) ↓, muscular lipid accumulation ↓, Muscle inflammation ↓	[171]
		Cachectic mice.	Muscle mass ↑, protein degradation (atrogin-1, MuRF1) ↓	[172]
Flavonol	Quercetin	HFD-fed ICR mice	Lipogenesis ↓, lipolysis ↑, WAT browning and thermogenesis ↑, AMPK ↑,	[180]
		HFD-fed aged mice	Body Weight ↓	[181]
		3T3-L1 cells	Adipogenesis ↓, AMPK ↑, apoptosis ↑	[182]
		HFD-fed C57BL/6J mice	Body Weight ↓, fat mass ↓, lipogenesis ↓	[183]
		Rat adipocytes	Lipolysis ↑	[184]
		Subjects with APOE genotype	Waist circumference ↓	[187]
		HFD-fed C57BL/6J mice	Fat mass ↓, Muscle mass ↑, muscle fiber size ↑, protein degradation (atrogin-1, MuRF1) ↓, Muscle inflammation ↓	[189]
		PA-treated cocultured C2C12 myotubes and Raw264.7 cells	Protein degradation (atrogin-1, MuRF1) ↓, inflammation ↓	[189]
		TNFα-treated myotubes	Protein degradation (atrogin-1, MAFbx, MuRF1) ↓, oxidative stress ↓, inflammation ↓	[190]
		HFD-fed C57BL/6J mice	Improvement in muscle atrophy, oxidative stress ↓, inflammation ↓	[190]
		Tail-suspension mice	Muscle mass ↑, protein degradation (atrogin-1, MuRF1) ↓, oxidative stress ↓	[191]
		Denervated mice	Muscle mass ↑, muscle fiber size ↑, protein synthesis (IGF-1, AKT) ↑	[192]
		Cachectic mice.	Muscle mass ↑, improvement in mitochondrial homeostatic balance	[193]
		Denervated mice	Improvement in muscle atrophy	[194]
flavonol	Dihydromyricetin	3T3-L1 cells	lipid droplet formation ↓, Adipogenesis ↓	[202]
		HFD-fed C57BL/6J mice	Body weight ↓, Fat mass ↓, WAT browning ↑	[203]
		ob/ob mice	Body weight gain ↓, gut microbiota regulation	[205]
		HFD-fed C57BL/6J mice, ob/ob mice	Muscle insulin resistance ↓, proportion of type I fibers ↓	[206]
		SD rats under simulated high-altitude conditions	Muscle function ↑, hypobaric hypoxia-induced mitochondrial dysfunction ↓, mitochondrial biogenesis ↑	[208]
		SD rats	Muscle mass ↑, muscle fiber size ↑, mitochondrial biogenesis ↑, improvement in mitochondrial dysfunction, oxidative stress ↓	[209]
		D-gal-induced aging rats	Muscle mass ↑, muscle fiber size ↑, protein degradation (atrogin-1, MAFbx) ↓, AMPK ↑	[210]
		HFD-fed C57BL/6J mice	Fat mass ↓, muscle mass ↑, inflammation ↓, muscle function ↑, protein degradation (atrogin-1) ↓, protein synthesis (mTOR) ↑, AMPK ↑	[211]
		TNF-α-treated C2C12 myotubes	Inflammation ↓, protein degradation (atrogin-1, MuRF1) ↓, protein synthesis (mTOR) ↑, AMPK ↑	[211]
flavanol	EGCG	HFD-fed C57BL/6J mice	Body weight gain ↓, fat mass ↓, BAT thermogenesis and mitochondrial biogenesis ↑, AMPK ↑	[219]
		HFD-fed C57BL/6J mice	Body weight ↓, fat mass ↓, BAT thermogenesis ↑	[220]
		HFD-fed aged mice	Body weight ↓, fat mass ↓, food intake ↑, lipogenesis ↓, fatty acid oxidation ↑, gut microbiota regulation	[221]
		HFD-fed C57BL/6J mice	Fat mass ↓, lipogenesis ↓, lipolysis ↑, AMPK ↑	[222]
		HFD-fed C57BL/6J mice	Body weight ↓, fat mass ↓, lipogenesis ↓, lipolysis ↑, autophagy ↑, AMPK ↑	[223]
		3T3-L1 cells	Adipogenesis ↓	[224,225]
		Overweight men	Fatty acid oxidation ↑	[226]
		Aged rats (muscle disuse as hindlimb unloading)	Muscle mass ↑, muscle fiber size ↑, muscle function ↑, apoptosis ↓, satellite proliferation ↑	[230]
		Aged rats (muscle recovery after forced disuse)	Recovery from disuse, autophagy ↓, apoptosis ↓	[231]
		Aged rats	Muscle mass ↑, muscle fiber size ↑, protein degradation (MuRF1, MAFbx) ↓, myostatin ↓, protein synthesis (IGF-1) ↑,	[232]
		Aged mice	Muscle mass ↑, protein synthesis (IGF-1, AKT) ↑, protein degradation (atrogin-1, FoxO, MuRF) ↓	[233]
		Cachectic mice	Muscle mass ↑, protein degradation (MuRF, MAFbx) ↓, inflammation ↓	[234]
		Denervated rats	Muscle function ↑, apoptosis ↓	[235]
		Mice	Endurance capacity ↑, fatty acid oxidation ↑	[236]
		GK rats	Oxidative stress ↓, mitochondrial dysfunction ↓, autophagy ↓	[237]
		C2C12 myotubes	Protein degradation (MuRF1, MAFbx) ↓, protein synthesis (AKT) ↑	[238]
flavanol	Epicatechin	Aged mice	Muscle fiber size ↑, muscle function ↑	[239]
		Obese middle-aged mice	Body weight ↓, fat mass ↓, muscle function ↑, protein degradation (MuRF) ↓, muscle growth and differentiation ↑, oxidative stress ↓	[240]
		Aged mice	Oxidative stress ↓, mitochondrial biogenesis ↑	[241]
		C2C12 myotubes	Protein degradation (atrogin-1, MuRF1) ↓	[242]
		C2C12 myotubes	Mitochondrial biogenesis ↑	[243]
		Sarcopenic older adults (resistance training and epicatechin supplementation)	Muscle mass ↑, Skeletal muscle strength ↑, myostatin ↓, muscle growth factors ↑	[244]
flavone	5,7-dimethoxyflavon	Aged mice	Muscle mass ↑, muscle fiber size ↑, muscle function ↑, protein synthesis (AKT, mTOR) ↑, protein degradation (atrogin-1, MuRF) ↓, mitochondrial biogenesis ↑, inflammation ↓	[247]
		3T3-L1 cell	Adipogenesis ↓	[248]
		HFD-fed C57BL/6J mice	Body weight gain ↓, fat mass ↓, adipogenesis ↓	[248]
isoflavan	Glabridin	Dexamethasone-treated C2C12 myotube	Protein degradation (MuRF1, FoxO) ↓,	[249]
		Dexamethasone-treated mice	Muscle mass ↑, protein degradation (MuRF1, FoxO) ↓,	[249]
		HFD-fed C57BL/6J mice	Body weight ↓, fat mass ↓, food intake ↓, energy expenditure ↑, inflammation ↓, lipogenesis ↓, fatty acid oxidation ↑, AMPK ↑	[250]
		C2C12 myotubes	Fatty acid oxidation ↑, AMPK ↑	[250]
flavanone	Hesperetin	HFD-fed C57BL/6J mice	Body weight gain ↓, fat mass ↓	[251]
		Aged mice	Muscle fiber size ↑, muscle function ↑, oxidative stress ↓	[252]
		Aged mice	Fat mass ↓, muscle mass ↑, muscle fiber size ↑, energy expenditure ↑	[253]
flavanone	naringenin	HFD-fed ovariectomized mice	Fat mass ↓, inflammation ↓	[254]
		Ovariectomized mice	Fat mass ↓, muscle mass ↑, muscle lipid accumulation ↓	[255]
		L6 myoblast, C2C12 myoblasts, satellite cells	Regulation of skeletal muscle differentiation	[256]
		HFHC diet-fed Ldlr−/− mice	Fat mass ↓, energy expenditure ↑, insulin resistance ↓	[257]

APOE, apolipoprotein E; D-gal, D-galactose; HFD, high-fat diet; HFHC, high fat and high cholesterol; LPS, lipopolysaccharide; PA, palmitic acid.

## Data Availability

Data are contained within the article.

## References

[1] WHO Obesity Report. World Health Organization. https://www.who.int/news-room/fact-sheets/detail/obesity-and-overweight.

[2] Nations U. World Population Prospects: The 2017 Revisions, Key Findings and Advance Tables. https://https://reliefweb.int/report/world/world-population-prospects-2017-revision-key-findings-and-advance-tables.

[3] United Nations Department of Economic and Social Affairs (DESA)/Population Division (2022). World Population Prospects. https://population.un.org/wpp/Download/Standard/Population..

[4] Kaeberlein M., Rabinovitch P.S., Martin G.M. (2015). Healthy aging: The ultimate preventative medicine. Science.

[5] Heber D., Ingles S., Ashley J.M., Maxwell M.H., Lyons R.F., Elashoff R.M. (1996). Clinical detection of sarcopenic obesity by bioelectrical impedance analysis. Am. J. Clin. Nutr..

[6] Purcell S.A., Mackenzie M., Barbosa-Silva T.G., Dionne I.J., Ghosh S., Siervo M., Ye M., Prado C.M. (2021). Prevalence of sarcopenic obesity using different definitions and the relationship with strength and physical performance in the Canadian Longitudinal Study of Aging. Front. Physiol..

[7] Alves Guimarães M.S., Araújo dos Santos C., da Silva Castro J., Juvanhol L.L., Canaan Rezende F.A., Martinho K.O., Ribeiro A.Q. (2021). Prevalence, diagnostic criteria, and factors associated with sarcopenic obesity in older adults from a low middle income country: A systematic review. Clin. Nutr. ESPEN.

[8] Molino S., Dossena M., Buonocore D., Verri M. (2016). Sarcopenic obesity: An appraisal of the current status of knowledge and management in elderly people. J. Nutr. Health Aging.

[9] Kim Y.S., Lee Y., Chung Y.S., Lee D.J., Joo N.S., Hong D., Song G.E., Kim H.J., Choi Y.J., Kim K.M. (2012). Prevalence of sarcopenia and sarcopenic obesity in the Korean population based on the Fourth Korean National Health and Nutritional Examination Surveys. J. Gerontol. A Biol. Sci. Med. Sci..

[10] Wagenaar C.A., Dekker L.H., Navis G.J. (2021). Prevalence of sarcopenic obesity and sarcopenic overweight in the general population: The lifelines cohort study. Clin. Nutr..

[11] Sack C., Ferrari N., Friesen D., Haas F., Klaudius M., Schmidt L., Torbahn G., Wulff H., Joisten C. (2022). Health risks of sarcopenic obesity in overweight children and adolescents: Data from the CHILT III programme (Cologne). J. Clin. Med..

[12] Jin K., Simpkins J.W., Ji X., Leis M., Stambler I. (2015). The critical need to promote research of aging and aging-related diseases to improve health and longevity of the elderly population. Aging Dis..

[13] Kim T.N., Choi K.M. (2013). Sarcopenic obesity. J. Korean Diabetes.

[14] Dalle S., Rossmeislova L., Koppo K. (2017). The role of inflammation in age-related sarcopenia. Front. Physiol..

[15] Jang H.C. (2011). Recent progression in sarcopenia and sarcopenic obesity. J. Korean Geriatr. Soc..

[16] Kalyani R.R., Corriere M., Ferrucci L. (2014). Age-related and disease-related muscle loss: The effect of diabetes, obesity, and other diseases. Lancet Diabetes Endocrinol..

[17] Gonzalez A., Simon F., Achiardi O., Vilos C., Cabrera D., Cabello-Verrugio C. (2021). The critical role of oxidative stress in sarcopenic obesity. Oxidative Med. Cell Longev..

[18] Diago-Galmés A., Guillamón-Escudero C., Tenías-Burillo J.M., Soriano J.M., Fernández-Garrido J. (2021). Salivary testosterone and cortisol as biomarkers for the diagnosis of sarcopenia and sarcopenic obesity in community-dwelling older adults. Biology.

[19] Alizadeh Pahlavani H. (2022). Exercise therapy for people with sarcopenic obesity: Myokines and adipokines as effective actors. Front. Endocrinol..

[20] Panche A.N., Diwan A.D., Chandra S.R. (2016). Flavonoids: An overview. J. Nutr. Sci..

[21] Boccellino M., D’Angelo S. (2020). Anti-obesity effects of polyphenol intake: Current status and future possibilities. Int. J. Mol. Sci..

[22] Kim C., Hwang J.K. (2020). Flavonoids: Nutraceutical potential for counteracting muscle atrophy. Food Sci. Biotechnol..

[23] Tandon P., Dunn M.A., Duarte-Rojo A. (2020). Resistance training reduces risk of sarcopenia in patients with cirrhosis. Clin. Gastroenterol. Hepatol..

[24] Wang H., Hai S., Cao L., Zhou J., Liu P., Dong B.R. (2016). Estimation of prevalence of sarcopenia by using a new bioelectrical impedance analysis in Chinese community-dwelling elderly people. BMC Geriatr..

[25] Santilli V., Bernetti A., Mangone M., Paoloni M. (2014). Clinical definition of sarcopenia. Clin. Cases Miner Bone Metab..

[26] Cruz-Jentoft J., Bahat G., Bauer J., Boirie Y., Bruyère O., Cederholm T., Cooper C., Landi F., Rolland Y., Sayer A.A. (2019). Writing Group for the European Working Group on Sarcopenia in Older People 2 (EWGSOP2), and the Extended Group for EWGSOP2. Sarcopenia: Revised European consensus on definition and diagnosis. Age Ageing.

[27] Keller K. (2019). Sarcopenia. Wien. Med. Wochenschr..

[28] Cawthon P.M., Manini T., Patel S.M., Newman A., Travison T., Kiel D.P., Santanasto A.J., Ensrud K.E., Xue Q.L., Shardell M. (2020). Putative cut-points in sarcopenia components and incident adverse health outcomes: An SDOC analysis observational study. J. Am. Geriatr. Soc..

[29] Sharma P., Zargar-Shoshtari K., Caracciolo J.T., Fishman M., Poch M.A., Pow-Sang J., Sexton W.J., Spiess P.E. (2015). Sarcopenia as a predictor of overall survival after cytoreductive nephrectomy for metastatic renal cell carcinoma. Urol. Oncol..

[30] Merli M., Giusto M., Lucidi C., Giannelli V., Pentassuglio I., Di Gregorio V., Lattanzi B., Riggio O. (2013). Muscle depletion increases the risk of overt and minimal hepatic encephalopathy: Results of a prospective study. Metab. Brain Dis..

[31] Mir O., Coriat R., Blanchet B., Durand J.P., Boudou-Rouquette P., Michels J., Ropert S., Vidal M., Pol S., Chaussade S. (2012). Sarcopenia predicts early dose-limiting toxicities and pharmacokinetics of sorafenib in patients with hepatocellular carcinoma. PLoS ONE.

[32] Cousin S., Hollebecque A., Koscielny S., Mir O., Varga A., Baracos V.E., Soria J.C., Antoun S. (2014). Low skeletal muscle is associated with toxicity in patients included in phase I. trials. Investig. New Drugs.

[33] Prado C.M.M., Baracos V.E., McCargar L.J., Reiman T., Mourtzakis M., Tonkin K., Mackey J.R., Koski S., Pituskin E., Sawyer M.B. (2009). Sarcopenia as a determinant of chemotherapy toxicity and time to tumor progression in metastatic breast cancer patients receiving capecitabine treatment. Clin. Cancer Res..

[34] Tan B.H.L., Birdsell L.A., Martin L., Baracos V.E., Fearon K.C.H. (2009). Sarcopenia in an overweight or obese patient is an adverse prognostic factor in pancreatic cancer. Clin. Cancer Res..

[35] Janssen I., Shepard D.S., Katzmarzyk P.T., Roubenoff R. (2004). The healthcare costs of sarcopenia in the United States. J. Am. Geriatr. Soc..

[36] Jayarathne S., Koboziev I., Park O.H., Oldewage-Theron W., Shen C.L., Moustaid-Moussa N. (2017). Anti-Inflammatory and Anti-Obesity Properties of Food Bioactive Components: Effects on Adipose Tissue. Prev. Nutr. Food Sci..

[37] World Health Organization IOTF (2000). The Asian-Pacific Perspective: Redefining Obesity and Its Treatment.

[38] Pi-Sunyer F.X., Becker D.M., Bouchard C., Carleton R.A., Colditz G.A., Dietz W.H. (1998). Expert Panel on the Identification, Evaluation, and Treatment of Overweight in Adults. Clinical guidelines on the identification, evaluation, and treatment of overweight and obesity in adults: Executive summary. Am. J. Clin. Nutr..

[39] Avgerinos K.I., Spyrou N., Mantzoros C.S., Dalamaga M. (2019). Obesity and cancer risk: Emerging biological mechanisms and perspectives. Metabolism.

[40] Issa R.I., Griffin T.M. (2012). Pathobiology of obesity and osteoarthritis: Integrating biomechanics and inflammation. Pathobiol. Aging Age Relat. Dis..

[41] Shen Y.C., Kung S.C., Chang E.T., Hong Y.L., Wang L.Y. (2019). The impact of obesity in cognitive and memory dysfunction in obstructive sleep apnea syndrome. Int. J. Obes..

[42] O’Rourke R.W., Lumeng C.N. (2021). Pathways to severe COVID-19 for people with obesity. Obesity.

[43] WHO Controlling the Global Obesity Epidemic. https://www.who.int/activities/controlling-the-global-obesity-epidemic.

[44] Malenfant J.H., Batsis J.A. (2019). Obesity in the geriatric population—A global health perspective. J. Glob. Health Rep..

[45] Hong S.H., Choi K.M. (2020). Sarcopenic obesity, insulin resistance, and their implications in cardiovascular and metabolic consequences. Int. J. Mol. Sci..

[46] Donini L.M., Busetto L., Bischoff S.C., Cederholm T., Ballesteros-Pomar M.D., Batsis J.A., Bauer J.M., Boirie Y., Cruz-Jentoft A.J., Dicker D. (2022). Definition and diagnostic criteria for sarcopenic obesity: ESPEN and EASO consensus statement. Clin. Nutr..

[47] Stephen W.C., Janssen I. (2009). Sarcopenic-obesity and cardiovascular disease risk in the elderly. J. Nutr. Health Aging.

[48] Lu C.W., Yang K.C., Chang H.H., Lee L.T., Chen C.Y., Huang K.C. (2013). Sarcopenic obesity is closely associated with metabolic syndrome. Obes. Res. Clin. Pract..

[49] Song W., Yoo S.H., Jang J., Baik S.J., Lee B.K., Lee H.W., Park J.S. (2023). Association between sarcopenic obesity status and nonalcoholic fatty liver disease and fibrosis. Gut Liver.

[50] Lin Y.H., Teng M.M.H. (2022). Association of possible sarcopenic obesity with osteoporosis and fragility fractures in postmenopausal women. Arch. Osteoporos..

[51] Kim H.I., Ahn S.H., Kim Y., Lee J.E., Choi E., Seo S.K. (2022). Effects of sarcopenia and sarcopenic obesity on joint pain and degenerative osteoarthritis in postmenopausal women. Sci. Rep..

[52] Lee S.E., Park J.H., Kim K.A., Kang Y.S., Choi H.S. (2020). Association between sarcopenic obesity and pulmonary function in Korean ederly: Results from the Korean National Health and Nutrition Examination Survey. Calcif. Tissue Int..

[53] Seo D.H., Suh Y.J., Cho Y., Ahn S.H., Seo S., Hong S., Lee Y.H., Choi Y.J., Lee E., Kim S.H. (2022). Effect of low skeletal muscle mass and sarcopenic obesity on chronic kidney disease in patients with type 2 diabetes. Obesity.

[54] Hamer M., Batty G.D., Kivimaki M. (2015). Sarcopenic obesity and risk of new onset depressive symptoms in older adults: English longitudinal study of ageing. Int. J. Obes..

[55] Ishii S., Chang C., Tanaka T., Kuroda A., Tsuji T., Akishita M., Iijima K. (2016). The association between sarcopenic obesity and depressive symptoms in older Japanese adults. PLoS ONE.

[56] Gruber E.S., Jomrich G., Tamandl D., Gnant M., Schindl M., Sahora K. (2019). Sarcopenia and sarcopenic obesity are independent adverse prognostic factors in resectable pancreatic ductal adenocarcinoma. PLoS ONE.

[57] Wilkinson T.J., Yates T., Baker L.A., Zaccardi F., Smith A.C. (2022). Sarcopenic obesity and the risk of hospitalization or death from coronavirus disease 2019: Findings from UK Biobank. JCSM Rapid Commun..

[58] Liguori I., Russo G., Curcio F., Bulli G., Aran L., Della-Morte D., Gargiulo G., Testa G., Cacciatore F., Bonaduce D. (2018). Oxidative stress, aging, and diseases. Clin. Interv. Aging.

[59] Meng S.J., Yu L.J. (2010). Oxidative stress, molecular inflammation and sarcopenia. Int. J. Mol. Sci..

[60] Cheng Z., Tseng Y., White M.F. (2010). Insulin signaling meets mitochondria in metabolism. Trends Endocrinol. Metab..

[61] David S., Hernández-Alvarez M.I., Segalés J., Sorianello E., Muñoz J.P., Sala D., Waget A., Liesa M., Paz J.C., Gopalacharyulu P. (2012). Mitofusin 2 (Mfn2) links mitochondrial and endoplasmic reticulum function with insulin signaling and is essential for normal glucose homeostasis. Proc. Natl. Acad. Sci. USA.

[62] Figueiredo P.A., Mota M.P., Appell H.J., Duarte J.A. (2008). The role of mitochondria in aging of skeletal muscle. Biogerontology.

[63] Rooyackers O.E., Adey D.B., Ades P.A., Nair K.S. (1996). Effect of age on in vivo rates of mitochondrial protein synthesis in human skeletal muscle. Proc. Natl. Acad. Sci. USA.

[64] Boffoli D., Scacco S.C., Vergari R., Solarino G., Santacroce G., Papa S. (1994). Decline with age of the respiratory chain activity in human skeletal muscle. Biochim. Biophys. Acta.

[65] Larsen R.G., Callahan D.M., Foulis S.A., Kent-Braun J.A. (2012). Age-related changes in oxidative capacity differ between locomotory muscles and are associated with physical activity behavior. Appl. Physiol. Nutr. Metab..

[66] Hyatt H.W., Powers S.K. (2021). Mitochondrial dysfunction is a common denominator linking skeletal muscle wasting due to disease, aging, and prolonged inactivity. Antioxidants.

[67] Daw C.K., Starnes J.W., White T.P. (1988). Muscle atrophy and hypoplasia with aging: Impact of training and food restriction. J. Appl. Physiol..

[68] Lexell J., Taylor C.C., Sjostrom M. (1988). What is the cause of the ageing atrophy? Total number, size and proportion of different fiber types studied in whole vastus lateralis muscle from 15- to 83-year-old men. J. Neurol. Sci..

[69] Lexell J. (1995). Human aging, muscle mass, and fiber type composition. J. Gerontol. A Biol. Sci. Med. Sci..

[70] Nilwik R., Snijders T., Leenders M., Groen B.B., van Kranenburg J., Verdijk L.B., van Loon L.J. (2013). The decline in skeletal muscle mass with aging is mainly attributed to a reduction in type II muscle fiber size. Exp. Gerontol..

[71] Kusminski C.M., Scherer P.E. (2012). Mitochondrial dysfunction in white adipose tissue. Trends Endocrinol. Metab..

[72] Boudina S., Graham T.E. (2014). Mitochondrial function/dysfunction in white adipose tissue. Exp. Physiol..

[73] Baldini F., Fabbri R., Eberhagen C., Voci A., Portincasa P., Zischka H., Vergani L. (2021). Adipocyte hypertrophy parallels alterations of mitochondrial status in a cell model for adipose tissue dysfunction in obesity. Life Sci..

[74] Liesa M., Shirihai O.S. (2013). Mitochondrial dynamics in the regulation of nutrient utilization and energy expenditure. Cell Metab..

[75] Jackisch L., Murphy A.M., Kumar S., Randeva H., Tripathi G., McTernan P.G. (2020). Tunicamycin-induced endoplasmic reticulum stress mediates mitochondrial dysfunction in human adipocytes. J. Clin. Endocrinol. Metab..

[76] O’Neill H.M., Holloway G.P., Steinberg G.R. (2013). AMPK regulation of fatty acid metabolism and mitochondrial biogenesis: Implications for obesity. Mol. Cell Endocrinol..

[77] Zhang J., Wang Y., Liu X., Dagda R.K., Zhang Y. (2017). How AMPK and PKA interplay to regulate mitochondrial function and survival in models of ischemia and diabetes. Oxidative Med. Cell Longev..

[78] Chong W.C., Shastri M.D., Eri R. (2017). Endoplasmic reticulum stress and oxidative stress: A vicious nexus implicated in bowel disease pathophysiology. Int. J. Mol. Sci..

[79] Tripathi Y.B., Pandey V. (2012). Obesity and endoplasmic reticulum (ER) stresses. Front. Immunol..

[80] Gregor M.F., Hotamisligil G.S. (2007). Adipocyte stress: The endoplasmic reticulum and metabolic disease. J. Lipid Res..

[81] Boden G., Duan X., Homko C., Molina E.J., Song W., Perez O., Cheung P., Merali S. (2008). Increase in endoplasmic reticulum stress-related proteins and genes in adipose tissue of obese insulin-resistant individuals. Diabetes.

[82] Ozcan U., Cao Q., Yilmaz E., Lee A.H., Iwakoshi N.N., Ozdelen E., Tuncman G., Görgün C., Glimcher L.H., Hotamisligil G.S. (2004). Endoplasmic reticulum stress links obesity, insulin action, and type 2 diabetes. Science.

[83] Zhang X., Zhang G., Zhang H., Karin M., Bai H., Cai D. (2008). Hypothalamic IKKbeta/NF-kappaB and ER stress link overnutrition to energy imbalance and obesity. Cell.

[84] Gregor M.F., Yang L., Fabbrini E., Mohammed B.S., Eagon J.C., Hotamisligil G.S., Klein S. (2009). Endoplasmic reticulum stress is reduced in tissues of obese subjects after weight loss. Diabetes.

[85] Ozcan U., Yilmaz E., Ozcan L., Furuhashi M., Vaillancourt E., Smith R.O., Görgün C.Z., Hotamisligil G.S. (2006). Chemical chaperones reduce ER stress and restore glucose homeostasis in a mouse model of type 2 diabetes. Science.

[86] Kawasaki N., Asada R., Saito A., Kanemoto S., Imaizumi K. (2012). besity-induced endoplasmic reticulum stress causes chronic inflammation in adipose tissue. Sci. Rep..

[87] Chalil S., Pierre N., Bakker A.D., Manders R.J., Pletsers A., Francaux M., Klein-Nulend J., Jaspers R.T., Deldicque L. (2015). Aging related ER stress is not responsible for anabolic resistance in mouse skeletal muscle. Biochem. Biophys. Res. Commun..

[88] Deldicque L. (2013). Endoplasmic reticulum stress in human skeletal muscle: Any contribution to sarcopenia?. Front. Physiol..

[89] Ogata T., Machida S., Oishi Y., Higuchi M., Muraoka I. (2009). Differential cell death regulation between adult-unloaded and aged rat soleus muscle. Mech. Ageing Dev..

[90] O’Leary M.F., Vainshtein A., Iqbal S., Ostojic O., Hood D.A. (2013). Adaptive plasticity of autophagic proteins to denervation in aging skeletal muscle. Am. J. Physiol. Cell Physiol..

[91] Bañuls C., Rovira-Llopis S., Lopez-Domenech S., Diaz-Morales N., Blas-Garcia A., Veses S., Morillas C., Victor V.M., Rocha M., Hernandez-Mijares A. (2017). Oxidative and endoplasmic reticulum stress is impaired in leukocytes from metabolically unhealthy vs healthy obese individuals. Int. J. Obes..

[92] Jiao G., Hao L., Wang M., Zhong B., Yu M., Zhao S., Wang P., Feng R., Tan S., Chen L. (2017). Upregulation of endoplasmic reticulum stress is associated with diaphragm contractile dysfunction in a rat model of sepsis. Mol. Med. Rep..

[93] Ibrahim Z., Ramachandran G., El-Huneidi W., Elmoselhi A., Qaisar R. (2022). Suppression of endoplasmic reticulum stress prevents disuse muscle atrophy in a mouse model of microgravity. Life Sci. Space Res..

[94] McCarthy J.J., Esser K.A. (2010). Anabolic and catabolic pathways regulating skeletal muscle mass. Curr. Opin. Clin. Nutr. Metab. Care.

[95] Canfora I., Tarantino N., Pierno S. (2022). Metabolic pathways and ion channels involved in skeletal muscle atrophy: A starting point for Potential Therapeutic Strategies. Cells.

[96] King D., Yeomanson D., Bryant H.E. (2015). PI3King the lock: Targeting the PI3K/Akt/mTOR pathway as a novel therapeutic strategy in neuroblastoma. J. Pediatr. Hematol. Oncol..

[97] Yoon M.S. (2017). mTOR as a key regulator in maintaining skeletal muscle mass. Front. Physiol..

[98] Kriete A., Bosl W.J., Booker G. (2010). Rule-based cell systems model of aging using feedback loop motifs mediated by stress responses. PLoS Comput. Biol..

[99] Anand A., Nambirajan A., Kumar V., Agarwal S., Sharma S., Mohta S., Gopi S., Kaushal K., Gunjan D., Singh N. (2022). Alterations in autophagy and mammalian target of rapamycin (mTOR) pathways mediate sarcopenia in patients with cirrhosis. J. Clin. Exp. Hepatol..

[100] Gyawali B., Shimokata T., Honda K., Kondoh C., Hayashi N., Yoshino Y., Sassa N., Nakano Y., Gotoh M., Ando Y. (2016). Muscle wasting associated with the long-term use of mTOR inhibitors. Mol. Clin. Oncol..

[101] Bodine S.C. (2006). mTOR signaling and the molecular adaptation to resistance exercise. Med. Sci. Sport. Exerc..

[102] Haddad F., Adams G.R. (2006). Aging-sensitive cellular and molecular mechanisms associated with skeletal muscle hypertrophy. J. Appl. Physoil..

[103] Bian A., Ma Y., Zhou X., Guo Y., Wang W., Zhang Y., Wang X. (2020). Association between sarcopenia and levels of growth hormone and insulin-like growth factor-1 in the elderly. BMC Musculoskelet. Disord..

[104] Kwak J.Y., Hwang H., Kim S.K., Choi J.Y., Lee S.M., Bang H., Kwon E.S., Lee K.P., Chung S.G., Kwon K.S. (2018). Prediction of sarcopenia using a combination of multiple serum biomarkers. Sci. Rep..

[105] Kubo H., Sawada S., Satoh M., Asai Y., Kodama S., Sato T., Tomiyama S., Seike J., Takahashi K., Kaneko K. (2022). Insulin-like growth factor-1 levels are associated with high comorbidity of metabolic disorders in obese subjects; a Japanese single-center, retrospective-study. Sci. Rep..

[106] Bark T.H., McNurlan M.A., Lang C.H., Garlick P.J. (1998). Increased protein synthesis after acute IGF-I or insulin infusion is localized to muscle in mice. Am. J. Physiol..

[107] Armandi A., Rosso C., Caviglia G.P., Ribaldone D.G., Bugianesi E. (2021). The impact of dysmetabolic sarcopenia among insulin sensitive tissues: A narrative review. Front. Endocrinol..

[108] Wang X., Hu Z., Hu J., Du J., Mitch W.E. (2006). Insulin resistance accelerates muscle protein degradation: Activation of the ubiquitin-proteasome pathway by defects in muscle cell signaling. Endocrinology.

[109] Katta A., Kundla S., Kakarla S.K., Wu M., Fannin J., Paturi S., Liu H., Addagarla H.S., Blough E.R. (2010). Impaired overload-induced hypertrophy is associated with diminished mTOR signaling in insulin-resistant skeletal muscle of the obese Zucker rat. Am. J. Physiol. Regul. Integr. Comp. Physiol..

[110] Zeng Z., Liang J., Wu L., Zhang H., Lv J., Chen N. (2020). Exercise-induced autophagy suppresses sarcopenia through Akt/mTOR and Akt/FoxO3a signal pathways and AMPK-mediated mitochondrial quality control. Front. Physiol..

[111] Sirago G., Picca A., Calvani R., Coelho-Júnior H.J., Marzetti E. (2022). Mammalian target of rapamycin (mTOR) signaling at the crossroad of muscle fiber fate in sarcopenia. Int. J. Mol. Sci..

[112] Chrienova Z., Nepovimova E., Kuca K. (2021). The role of mTOR in age-related diseases. J. Enzym. Inhib. Med. Chem..

[113] Yoshida T., Tabony A.M., Galvez S., Mitch W.E., Higashi Y., Sukhanov S., Delafontaine P. (2013). Molecular mechanisms and signaling pathways of angiotensin II-induced muscle wasting: Potential therapeutic targets for cardiac cachexia. Int. J. Biochem. Cell Biol..

[114] Sandri M., Sandri C., Gilbert A., Skurk C., Calabria E., Picard A., Walsh K., Schiaffino S., Lecker S.H., Goldberg A.L. (2004). Foxo transcription factors induce the atrophy-related ubiquitin ligase atrogin-1 and cause skeletal muscle atrophy. Cell.

[115] Stitt T.N., Drujan D., Clarke B.A., Panaro F., Timofeyva Y., Kline W.O., Gonzalez M., Yancopoulos G.D., Glass D.J. (2004). The IGF-1/PI3K/Akt pathway prevents expression of muscle atrophy-induced ubiquitin ligases by inhibiting FOXO transcription factors. Mol. Cell.

[116] Mammucari C., Milan G., Romanello V., Masiero E., Rudolf R., Del Piccolo P., Burden S.J., Di Lisi R., Sandri C., Zhao J. (2007). FoxO3 controls autophagy in skeletal muscle in vivo. Cell Metab..

[117] Yang W., Zhang Y., Li Y., Wu Z., Zhu D. (2007). Myostatin induces cyclin D1 degradation to cause cell cycle arrest through a phosphatidylinositol 3-kinase/AKT/GSK-3 beta pathway and is antagonized by insulin-like growth factor 1. J. Biol. Chem..

[118] Chen N., Karantza-Wadsworth V. (2009). Role and regulation of autophagy in cancer. Biochim. Biophys. Acta.

[119] Terman A., Brunk U.T. (2006). Oxidative stress, accumulation of biological “garbage,” and aging. Antioxid. Redox Signal..

[120] Bonaldo P., Sandri M. (2013). Cellular and molecular mechanisms of muscle atrophy. Dis. Model. Mech..

[121] Pomiès P., Blaquière M., Maury J., Mercier J., Gouzi F., Hayot M. (2016). Involvement of the FoxO1/MuRF1/Atrogin-1 signaling pathway in the oxidative stress-induced atrophy of cultured chronic obstructive pulmonary disease myotubes. PLoS ONE.

[122] Kongara S., Karantza V. (2012). The interplay between autophagy and ROS in tumorigenesis. Front. Oncol..

[123] Li H., Malhotra S., Kumar A. (2008). Nuclear factor-kappa B signaling in skeletal muscle atrophy. J. Mol. Med..

[124] Bruunsgaard H., Pedersen B.K. (2003). Age-related inflammatory cytokines and disease. Immunol. Allergy Clin. N. Am..

[125] Cuthbertson D., Smith K., Babraj J., Leese G., Waddell T., Atherton P., Wackerhage H., Taylor P.M., Rennie M.J. (2005). Anabolic signaling deficits underlie amino acid resistance of wasting, aging muscle. FASEB J..

[126] Popko K., Gorska E., Stelmaszczyk-Emmel A., Plywaczewski R., Stoklosa A., Gorecka D., Pyrzak B., Demkow U. (2010). Proinflammatory cytokines Il-6 and TNF-α and the development of inflammation in obese subjects. Eur. J. Med. Res..

[127] Green C.J., Pedersen M., Pedersen B.K., Scheele C. (2011). Elevated NF-κB activation is conserved in human myocytes cultured from obese type 2 diabetic patients and attenuated by AMP-activated protein kinase. Diabetes.

[128] Snijders T., Nederveen J.P., McKay B.R., Joanisse S., Verdijk L.B., van Loon L.J., Parise G. (2015). Satellite cells in human skeletal muscle plasticity. Frontiers in physiology. Front. Physiol..

[129] Verdijk L.B., Snijders T., Drost M., Delhaas T., Kadi F., van Loon L.J.C. (2014). Satellite cells in human skeletal muscle; from birth to old age. Age.

[130] Roth S.M., Martel G.F., Ivey F.M., Lemmer J.T., Metter E.J., Hurley B.F., Rogers M.A. (2000). Skeletal muscle satellite cell populations in healthy young and older men and women. Anat. Rec..

[131] Wagers A.J., Conboy I.M. (2005). Cellular and molecular signatures of muscle regeneration: Current concepts and controversies in adult myogenesis. Cell.

[132] Van der Meer S.F.T., Jaspers R.T., Jones D.A., Degens H. (2011). Time-course of changes in the myonuclear domain during denervation in young-adult and old rat gastrocnemius muscle. Muscle Nerve.

[133] Huo F., Liu Q., Liu H. (2022). Contribution of muscle satellite cells to sarcopenia. Front. Physiol..

[134] Thornell L.E. (2011). Sarcopenic obesity: Satellite cells in the aging muscle. Curr. Opin. Clin. Nutr. Metab. Care.

[135] Beccafico S., Puglielli C., Pietrangelo T., Bellomo R., FanÒ G., Fulle S. (2007). Age-dependent effects on functional aspects in human satellite cells. Ann. N. Y. Acad. Sci..

[136] Minet A.D., Gaster M. (2012). Cultured senescent myoblasts derived from human vastus lateralis exhibit normal mitochondrial ATP synthesis capacities with correlating concomitant ROS production while whole cell ATP production is decreased. Biogerontology.

[137] Fulle S., Didonna S., Puglielli C. (2005). Age-dependent imbalance of the antioxidative system in human satellite cells. Exp. Gerontol..

[138] García-Prat L., Martínez-Vicente M., Perdiguero E., Ortet L., Rodríguez-Ubreva J., Rebollo E., Ruiz-Bonilla V., Gutarra S., Ballestar E., Serrano A.L. (2016). Autophagy maintains stemness by preventing senescence. Nature.

[139] Szentesi P., Csernoch L., Dux L., Keller-Pintér A. (2019). Changes in redox signaling in the skeletal muscle with aging. Oxidative Med. Cell Longev..

[140] García-Prat L., Muñoz-Cánoves P. (2017). Aging, metabolism and stem cells: Spotlight on muscle stem cells. Mol. Cell Endocrinol..

[141] Brack A.S., Conboy M.J., Roy S., Lee M., Kuo C.J., Keller C., Rando T.A. (2007). Increased Wnt signaling during aging alters muscle stem cell fate and increases fibrosis. Science.

[142] Conboy I.M., Conboy M.J., Smythe G.M., Rando T.A. (2003). Notch-mediated restoration of regenerative potential to aged muscle. Science.

[143] Bernet J.D., Doles J.D., Hall J.K., Kelly Tanaka K., Carter T.A., Olwin B.B. (2014). p38 MAPK signaling underlies a cell-autonomous loss of stem cell self- renewal in skeletal muscle of aged mice. Nat. Med..

[144] Tierney M.T., Aydogdu T., Sala D., Malecova B., Gatto S., Puri P.L., Latella L., Sacco A. (2014). STAT3 signaling controls satellite cell expansion and skeletal muscle repair. Nat. Med..

[145] Shirakawa T., Toyono T., Inoue A., Matsubara T., Kawamoto T., Kokabu S. (2022). Factors regulating or regulated by myogenic regulatory factors in skeletal muscle stem cells. Cells.

[146] Baig M.H., Ahmad K., Moon J.S., Park S.Y., Ho Lim J., Chun H.J., Qadri A.F., Hwang Y.C., Jan A.T., Ahmad S.S. (2022). Myostatin and its regulation: A comprehensive review of myostatin inhibiting strategies. Front. Physiol..

[147] Ryan A.S., Li G. (2021). Skeletal muscle myostatin gene expression and sarcopenia in overweight and obese middle-aged and older adults. JCSM Clin. Rep..

[148] White T.A., LeBrasseur N.K. (2014). Myostatin and sarcopenia: Opportunities and challenges—A mini-review. Gerontology.

[149] Allen D.L., Hittel D.S., McPherron A.C. (2011). Expression and function of myostatin in obesity, diabetes, and exercise adaptation. Med. Sci. Sport. Exerc..

[150] LeBrasseur N.K., Walsh K., Arany Z. (2011). Metabolic benefits of resistance training and fast glycolytic skeletal muscle. Am. J. Physiol. Endocrinol Metab..

[151] Akpan I., Goncalves M.D., Dhir R., Yin X., Pistilli E.E., Bogdanovich S., Khurana T.S., Ucran J., Lachey J., Ahima R.S. (2009). The effects of a soluble activin type IIB receptor on obesity and insulin sensitivity. Int. J. Obes..

[152] Bernardo B.L., Wachtmann T.S., Cosgrove P.G., Kuhn M., Opsahl A.C., Judkins K.M., Freeman T.B., Hadcock J.R., LeBrasseur N.K. (2010). Postnatal PPARdelta activation and myostatin inhibition exert distinct yet complimentary effects on the metabolic profile of obese insulin-resistant mice. PLoS ONE.

[153] Rocchetti G., Gregorio R.P., Lorenzo J.M., Barba F.J., Oliveira P.G., Prieto M.A., Simal-Gandara J., Mosele J.I., Motilva M.J., Tomas M. (2022). Functional implications of bound phenolic compounds and phenolics-food interaction: A review. Compr. Rev. Food Sci. Food Saf..

[154] Harborne J.B., Williams C.A. (2000). Advances in flavonoid research since 1992. Phytochemistry.

[155] Hostetler G.L., Ralston R.A., Schwartz S.J. (2017). Flavones: Food sources, bioavailability, metabolism, and bioactivity. Adv. Nutr..

[156] Ren B., Qin W., Wu F., Wang S., Pan C., Wang L., Zeng B., Ma S., Liang J. (2016). Apigenin and naringenin regulate glucose and lipid metabolism, and ameliorate vascular dysfunction in type 2 diabetic rats. Eur. J. Pharm..

[157] Shukla S., Bhaskaran N., Babcook M.A., Fu P., Maclennan G.T., Gupta S. (2014). Apigenin inhibits prostate cancer progression in TRAMP mice via targeting PI3K/Akt/FoxO pathway. Carcinogenesis.

[158] Huang C.H., Kuo P.L., Hsu Y.L., Chang T.T., Tseng H.I., Chu Y.T., Kuo C.H., Chen H.N., Hung C.H. (2010). The natural flavonoid apigenin suppresses Th1- and Th2-related chemokine production by human monocyte THP-1 cells through mitogen-activated protein kinase pathways. J. Med. Food.

[159] Siddique Y.H., Rahul Ara G., Afzal M., Varshney H., Gaur K., Subhan I., Mantasha I., Shahid M. (2022). Beneficial effects of apigenin on the transgenic Drosophila model of Alzheimer’s disease. Chem. Biol. Interact..

[160] Liu D., Peng R., Chen Z., Yu H., Wang S., Dong S., Li W., Shao W., Dai J., Li F. (2022). The protective effects of apigenin against radiation-induced intestinal injury. Dose Response.

[161] Jung U.J., Cho Y.Y., Choi M.S. (2016). Apigenin ameliorates dyslipidemia, hepatic steatosis and insulin resistance by modulating metabolic and transcriptional profiles in the liver of high-fat diet-induced obese mice. Nutrients.

[162] Myoung H.J., Kim G., Nam K.W. (2010). Apigenin isolated from the seeds of Perilla frutescens britton var crispa (Benth.) inhibits food intake in C57BL/6J mice. Arch. Pharm. Res..

[163] Sun Y.S., Qu W. (2019). Dietary apigenin promotes lipid catabolism, thermogenesis, and browning in adipose tissues of HFD-Fed mice. Food Chem. Toxicol..

[164] Qiao Y., Zhang Z., Zhai Y., Yan X., Zhou W., Liu H., Guan L., Peng L. (2022). Apigenin alleviates obesity-associated metabolic syndrome by regulating the composition of the gut microbiome. Front. Microbiol..

[165] Shiota C., Abe T., Kawai N., Ohno A., Teshima-Kondo S., Mori H., Terao J., Tanaka E., Nikawa T. (2015). Flavones inhibit LPS-induced atrogin-1/MAFbx expression in mouse C2C12 skeletal myotubes. J. Nutr. Sci. Vitaminol..

[166] Jang Y.J., Son H.J., Choi Y.M., Ahn J., Jung C.H., Ha T.Y. (2017). Apigenin enhances skeletal muscle hypertrophy and myoblast differentiation by regulating Prmt7. Oncotarget.

[167] Choi W.H., Jang Y.J., Son H.J., Ahn J., Jung C.H., Ha T.Y. (2018). Apigenin inhibits sciatic nerve denervation-induced muscle atrophy. Muscle Nerve.

[168] Choi W.H., Son H.J., Jang Y.J., Ahn J., Jung C.H., Ha T.Y. (2017). Apigenin ameliorates the obesity-induced skeletal muscle atrophy by attenuating mitochondrial dysfunction in the muscle of obese mice. Mol. Nutr. Food Res..

[169] Wang D., Yang Y., Zou X., Zhang J., Zheng Z., Wang Z. (2020). Antioxidant apigenin relieves age-related muscle atrophy by inhibiting oxidative stress and hyperactive mitophagy and apoptosis in skeletal muscle of mice. J. Gerontol. A Biol. Sci. Med. Sci..

[170] López-Lázaro M. (2009). Distribution and biological activities of the flavonoid luteolin. Mini Rev. Med. Chem..

[171] Kim J.W., Shin S.K., Kwon E.Y. (2023). Luteolin protects against obese sarcopenia in mice with high-fat diet-induced obesity by ameliorating inflammation and protein degradation in muscles. Mol. Nutr. Food Res..

[172] Chen T., Li B., Xu Y., Meng S., Wang Y., Jiang Y. (2018). Luteolin reduces cancer-induced skeletal and cardiac muscle atrophy in a Lewis lung cancer mouse model. Oncol. Rep..

[173] Anand David A.V., Arulmoli R., Parasuraman S. (2016). Overviews of biological importance of quercetin: A bioactive flavonoid. Pharmacogn. Rev..

[174] Braun K.F., Ehnert S., Freude T., Egana J.T., Schenck T.L., Buchholz A., Schmitt A., Siebenlist S., Schyschka L., Neumaier M. (2011). Quercetin protects primary human osteoblasts exposed to cigarette smoke through activation of the antioxidative enzymes HO-1 and SOD-1. Sci. World J..

[175] Maurya A.K., Vinayak M. (2015). Modulation of PKC signaling and induction of apoptosis through suppression of reactive oxygen species and tumor necrosis factor receptor 1 (TNFR1): Key role of quercetin in cancer prevention. Tumour. Biol..

[176] Li B., Yang M., Liu J.W., Yin G.T. (2016). Protective mechanism of quercetin on acute myocardial infarction in rats. Genet. Mol. Res..

[177] Hashemzaei M., Fanoudi S., Najari M., Fotouhi M., Belaran M., Alipour N.S., Dadrezaei Z., Miri F., Tabrizian K. (2021). Effects of quercetin and resveratrol on zinc chloride- and sodium metavanadate-induced passive avoidance memory retention deficits in male mice. Prev. Nutr. Food Sci..

[178] Moon J., Do H.J., Kim O.Y., Shin M.J. (2013). Antiobesity effects of quercetin-rich onion peel extract on the differentiation of 3T3-L1 preadipocytes and the adipogenesis in high fat-fed rats. Food Chem. Toxicol..

[179] Stewart L.K., Soileau J.L., Ribnicky D., Wang Z.Q., Raskin I., Poulev A., Majewski M., Cefalu W.T., Gettys T.W. (2008). Quercetin transiently increases energy expenditure but persistently decreases circulating markers of inflammation in C57BL/6J mice fed a high-fat diet. Metabolism.

[180] Jiang H., Horiuchi Y., Hironao K.Y., Kitakaze T., Yamashita Y., Ashida H. (2020). Prevention effect of quercetin and its glycosides on obesity and hyperglycemia through activating AMPKα in high-fat diet-fed ICR mice. J. Clin. Biochem. Nutr..

[181] Liang C., Oest M.E., Prater M.R. (2009). Intrauterine exposure to high saturated fat diet elevates risk of adult-onset chronic diseases in C57BL/6 mice. Birth Defects Res. B Dev. Reprod. Toxicol..

[182] Ahn J., Lee H., Kim S., Park J., Ha T. (2008). The anti-obesity effect of quercetin is mediated by the AMPK and MAPK signaling pathways. Biochem. Biophys. Res. Commun..

[183] Jung C.H., Cho I., Ahn J., Jeon T.I., Ha T.Y. (2013). Quercetin Reduces High-Fat Diet-Induced Fat Accumulation in the Liver by Regulating Lipid Metabolism Genes. Phytother. Res..

[184] Kuppusamy U.R., Das N.P. (1992). Effects of flavonoids on cyclic AMP phosphodiesterase and lipid mobilization in rat adipocytes. Biochem. Pharmacol..

[185] Kobori M., Takahashi Y., Sakurai M., Akimoto Y., Tsushida T., Oike H., Ippoushi K. (2016). Quercetin suppresses immune cell accumulation and improves mitochondrial gene expression in adipose tissue of diet-induced obese mice. Mol. Nutr. Food Res..

[186] Lee J.S., Cha Y.J., Lee K.H., Yim J.E. (2016). Onion peel extract reduces the percentage of body fat in overweight and obese subjects: A 12-week, randomized, double-blind, placebo-controlled study. Nutr. Res. Pract..

[187] Pfeuffer M., Auinger A., Bley U., Kraus-Stojanowic I., Laue C., Winkler P., Rüfer C.E., Frank J., Bösch-Saadatmandi C., Rimbach G. (2013). Effect of quercetin on traits of the metabolic syndrome, endothelial function and inflammation in men with different APOE isoforms. Nutr. Metab. Cardiovasc. Dis..

[188] Shanely R.A., Knab A.M., Nieman D.C., Jin F., McAnulty S.R., Landram M.J. (2010). Quercetin supplementation does not alter antioxidant status in humans. Free Radic. Res..

[189] Le N.H., Kim C.S., Park T., Yoon Park J.H., Sung M.K., Lee D.G., Hong S.M., Choe S.Y., Goto T., Kawada T. (2014). Quercetin protects against obesity-induced skeletal muscle inflammation and atrophy. Mediat. Inflamm..

[190] Kim Y., Kim C.S., Joe Y., Chung H.T., Ha T.Y., Yu R. (2018). Quercetin reduces tumor necrosis factor alpha-induced muscle atrophy by upregulation of heme oxygenase-1. J. Med. Food.

[191] Mukai R., Nakao R., Yamamoto H., Nikawa T., Takeda E., Terao J. (2010). Quercetin prevents unloading-derived disused muscle atrophy by attenuating the induction of ubiquitin ligases in tailsuspension mice. J. Nat. Prod..

[192] Mukai R., Matsui N., Fujikura Y., Matsumoto N., Hou D.X., Kanzaki N., Shibata H., Horikawa M., Iwasa K., Hirasaka K. (2016). Preventive effect of dietary quercetin on disuse muscle atrophy by targeting mitochondria in denervated mice. J. Nutr. Biochem..

[193] VanderVeen B.N., Cardaci T.D., Cunningham P., McDonald S.J., Bullard B.M., Fan D., Murphy E.A., Velázquez K.T. (2022). Quercetin improved muscle mass and mitochondrial content in a murine model of cancer and chemotherapy-induced cachexia. Nutrients.

[194] Chen M.M., Qin J., Chen S.J., Yao L.M., Zhang L.Y., Yin Z.Q., Liao H. (2017). Quercetin promotes motor and sensory function recovery following sciatic nerve-crush injury in C57BL/6J mice. J. Nutr. Biochem..

[195] Otsuka Y., Miyamoto N., Nagai A., Izumo T., Nakai M., Fukuda M., Arimitsu T., Yamada Y., Hashimoto T. (2022). Effects of quercetin glycoside supplementation combined with low-intensity resistance training on muscle quantity and stiffness: A randomized, controlled trial. Front. Nutr..

[196] Liu D., Mao Y., Ding L., Zeng X.A. (2019). Dihydromyricetin: A review on identification and quantification methods, biological activities, chemical stability, metabolism and approaches to enhance its bioavailability. Trends Food Sci. Technol..

[197] Ye L., Wang H., Duncan S.E., Eigel W.N., O’Keefe S.F. (2015). Antioxidant activities of Vine Tea (*Ampelopsis grossedentata*) extract and its major component dihydromyricetin in soybean oil and cooked ground beef. Food Chem..

[198] Hou X.L., Tong Q., Wang W.Q., Shi C.Y., Xiong W., Chen J., Liu X., Fang J.G. (2015). Suppression of inflammatory responses by dihydromyricetin, a flavonoid from ampelopsis grossedentata, via inhibiting the activation of NF-kappaB and MAPK signaling pathways. J. Nat. Prod..

[199] Zhao Z., Yin J.Q., Wu M.S., Song G., Xie X.B., Zou C., Tang Q., Wu Y., Lu J., Wang Y. (2014). Dihydromyricetin activates AMP-activated protein kinase and P38(MAPK) exerting antitumor potential in osteosarcoma. Cancer Prev. Res..

[200] Liu P., Zou D., Chen K., Zhou Q., Gao Y., Huang Y., Zhu J., Zhang Q., Mi M. (2016). Dihydromyricetin improves hypobaric Hypoxia-Induced memory impairment via modulation of SIRT3 signaling. Mol. Neurobiol..

[201] Qiu P., Dong Y., Li B., Kang X.J., Gu C., Zhu T., Luo Y.Y., Pang M.X., Du W.F., Ge W.H. (2017). Dihydromyricetin modulates p62 and autophagy crosstalk with the Keap-1/Nrf2 pathway to alleviate ethanol-induced hepatic injury. Toxicol. Lett..

[202] Sun B., Tan D., Pan D., Baker M.R., Liang Z., Wang Z., Lei J., Liu S., Hu C.Y., Li Q.X. (2021). Dihydromyricetin Imbues Antiadipogenic Effects on 3T3-L1 Cells via Direct Interactions with 78-kDa Glucose-Regulated Protein. J. Nutr..

[203] Leng Q., Zhou J., Li C., Xu Y., Liu L., Zhu Y., Yang Y., Zhang H., Li X. (2022). Dihydromyricetin ameliorates diet-induced obesity and promotes browning of white adipose tissue by upregulating IRF4/PGC-1α. Nutr. Metab..

[204] Wu J., Miyasaka K., Yamada W., Takeda S., Shimizu N., Shimoda H. (2022). The Anti-adiposity mechanisms of ampelopsin and vine tea extract in high fat diet and alcohol-induced fatty liver mouse models. Molecules.

[205] Song Y., Sun L., Ma P., Xu L., Xiao P. (2022). Dihydromyricetin prevents obesity via regulating bile acid metabolism associated with the farnesoid X receptor in ob/ob mice. Food Funct..

[206] Zhou Q., Gu Y., Lang H., Wang X., Chen K., Gong X., Zhou M., Ran L., Zhu J., Mi M. (2017). Dihydromyricetin prevents obesity-induced slow-twitch-fiber reduction partially via FLCN/FNIP1/AMPK pathway. Biochim. Biophys. Acta Mol. Basis Dis..

[207] Schiaffino S., Reggiani C. (2011). Fiber types in mammalian skeletal muscles. Physiol. Rev..

[208] Zou D., Chen K., Liu P., Chang H., Zhu J., Mi M. (2014). Dihydromyricetin improves physical performance under simulated high altitude. Med. Sci. Sport. Exerc..

[209] Huang Y., Chen K., Ren Q., Yi L., Zhu J., Zhang Q., Mi M. (2018). Dihydromyricetin attenuates dexamethasone-induced muscle atrophy by improving mitochondrial function via the PGC-1α pathway. Cell Physiol. Biochem..

[210] Kou X., Li J., Liu X., Yang X., Fan J., Chen N. (2017). Ampelopsin attenuates the atrophy of skeletal muscle from d-gal-induced aging rats through activating AMPK/SIRT1/PGC-1α signaling cascade. Biomed. Pharmacother..

[211] Hou L., Jiang F., Huang B., Zheng W., Jiang Y., Cai G., Liu D., Hu C.Y., Wang C. (2021). Dihydromyricetin resists inflammation-induced muscle atrophy via ryanodine receptor-CaMKK-AMPK signal pathway. J. Cell Mol. Med..

[212] Liu M., Tian H.L., Wu J.H., Cang R.R., Wang R.X., Qi X.H., Xu Q., Chen X.H. (2015). Relationship between gene expression and the accumulation of catechin during spring and autumn in tea plants (*Camellia sinensis* L.). Hortic. Res..

[213] Vrânceanu M., Galimberti D., Banc R., Dragoş O., Cozma-Petruţ A., Hegheş S.C., Voştinaru O., Cuciureanu M., Stroia C.M., Miere D. (2022). The anticancer potential of plant-derived nutraceuticals via the modulation of gene expression. Plants.

[214] Zhou D., Sun M.H., Jiang W.J., Li X.H., Lee S.H., Heo G., Niu Y.J., Ock S.A., Cui X.S. (2022). Epigallocatechin-3-gallate protects porcine oocytes against post-ovulatory aging through inhibition of oxidative stress. Aging.

[215] Wu Z., Shen J., Xu Q., Xiang Q., Chen Y., Lv L., Zheng B., Wang Q., Wang S., Li L. (2022). Epigallocatechin-3-gallate improves intestinal gut microbiota homeostasis and ameliorates clostridioides difficile Infection. Nutrients.

[216] Mou Q., Jia Z., Luo M., Liu L., Huang X., Quan J., Tian J. (2022). Epigallocatechin-3-gallate exerts cardioprotective effects related to energy metabolism in pressure overload-induced cardiac dysfunction. Arch. Biochem. Biophys..

[217] Soussi A., Gargouri M., Magné C., Ben-Nasr H., Kausar M.A., Siddiqui A.J., Saeed M., Snoussi M., Adnan M., El-Feki A. (2022). Epigallocatechin gallate (EGCG) pharmacokinetics and molecular interactions towards amelioration of hyperglycemia, hyperlipidemia associated hepatorenal oxidative injury in alloxan induced diabetic mice. Chem. Biol. Interact..

[218] Seok J.H., Kim D.H., Kim H.J., Jo H.H., Kim E.Y., Jeong J.H., Park Y.S., Lee S.H., Kim D.J., Nam S.Y. (2022). Epigallocatechin-3-gallate suppresses hemin-aggravated colon carcinogenesis through Nrf2-inhibited mitochondrial reactive oxygen species accumulation. J. Vet. Sci..

[219] Lee M.S., Shin Y., Jung S., Kim Y. (2017). Effects of epigallocatechin-3-gallate on thermogenesis and mitochondrial biogenesis in brown adipose tissues of diet-induced obese mice. Food Nutr. Res..

[220] Zhou J., Mao L., Xu P., Wang Y. (2018). Effects of (-)-epigallocatechin gallate (EGCG) on energy expenditure and microglia-mediated hypothalamic inflammation in mice fed a high-fat diet. Nutrients.

[221] Sheng L., Jena P.K., Liu H.X., Hu Y., Nagar N., Bronner D.N., Settles M.L., Bäumler A.J., Wan Y.Y. (2018). Obesity treatment by epigallocatechin-3-gallate-regulated bile acid signaling and its enriched *Akkermansia muciniphila*. FASEB J..

[222] Li F., Gao C., Yan P., Zhang M., Wang Y., Hu Y., Wu X., Wang X., Sheng J. (2018). EGCG reduces obesity and white adipose tissue gain partly through ampk activation in mice. Front. Pharmacol..

[223] Choi C., Song H.D., Son Y., Cho Y.K., Ahn S.Y., Jung Y.S., Yoon Y.C., Kwon S.W., Lee Y.H. (2020). Epigallocatechin-3-gallate reduces visceral adiposity partly through the regulation of beclin1-dependent autophagy in white adipose tissues. Nutrients.

[224] Moon H.S., Chung C.S., Lee H.G., Kim T.G., Choi Y.J., Cho C.S. (2007). Inhibitory effect of (-)-epigallocatechin-3-gallate on lipid accumulation of 3t3-l1 cells. Obesity.

[225] Wu M., Liu D., Zeng R., Xian T., Lu Y., Zeng G., Sun Z., Huang B., Huang Q. (2017). Epigallocatechin-3-gallate inhibits adipogenesis through down-regulation of PPARγ and FAS expression mediated by PI3K-AKT signaling in 3T3-L1 cells. Eur. J. Pharmacol..

[226] Boschmann M., Thielecke F. (2007). The effects of epigallocatechin-3-gallate on thermogenesis and fat oxidation in obese men: A pilot study. J. Am. Coll. Nutr..

[227] Mielgo-Ayuso J., Barrenechea L., Alcorta P., Larrarte E., Margareto J., Labayen I. (2014). Effects of dietary supplementation with epigallocatechin-3-gallate on weight loss, energy homeostasis, cardiometabolic risk factors and liver function in obese women: Randomised, double-blind, placebo-controlled clinical trial. Br. J. Nutr..

[228] Suzuki T., Pervin M., Goto S., Isemura M., Makamura Y. (2016). Beneficial effects of tea and the green tea catechin epigallocatechin-3-gallate on obesity. Molecules.

[229] Chatree S., Sitticharoon C., Maikaew P., Pongwattanapakin K., Keadkraichaiwat I., Churintaraphan M., Sripong C., Sririwichitchai R., Tapechum S. (2021). Epigallocatechin gallate decreases plasma triglyceride, blood pressure, and serum kisspeptin in obese human subjects. Exp. Biol. Med..

[230] Alway S.E., Bennett B.T., Wilson J.C., Edens N.K., Pereira S.L. (2014). Epigallocatechin-3-gallate improves plantaris muscle recovery after disuse in aged rats. Exp. Gerontol..

[231] Takahashi H., Suzuki Y., Mohamed J.S., Gotoh T., Pereira S.L., Always S.E. (2017). Epigallocatechin-3-gallate increases autophagy signaling in resting and unloaded plantaris muscles but selectively suppresses autophagy protein abundance in reloaded muscles of aged rats. Exp. Gerontol..

[232] Meador B.M., Mirza K.A., Tian M., Skelding M.B., Reaves L.A., Edens N.K., Tisdale M.J., Pereira S.L. (2015). The green tea polyphenol epigallocatechin-3-gallate (EGCG) attenuates skeletal muscle atrophy in a rat model of sarcopenia. J. Frailty Aging.

[233] Chang Y.C., Liu H.W., Chan Y.C., Hu S.H., Liu M.Y., Chang S.J. (2020). The green tea polyphenol epigallocatechin-3-gallate attenuates age-associated muscle loss via regulation of miR-486-5p and myostatin. Arch. Biochem. Biophys..

[234] Wang H., Lai Y.J., Chan Y.L., Li T.L., Wu C.J. (2011). Epigallocatechin-3-gallate effectively attenuates skeletal muscle atrophy caused by cancer cachexia. Cancer Lett..

[235] Renno W.M., Al-Maghrebi M., Al-Banaw A. (2012). (-)-Epigallocatechin-3-gallate (EGCG) attenuates functional deficits and morphological alterations by diminishing apoptotic gene overexpression in skeletal muscles after sciatic nerve crush injury. Naunyn. Schmiedebergs Arch. Phamacol..

[236] Murase T., Haramizu S., Shimotoyodome A., Nagasawa A., Tokimitsu I. (2005). Green tea extract improves endurance capacity and increases muscle lipid oxidation in mice. Am. J. Physiol. Regul. Integr. Comp. Physiol..

[237] Yan J., Feng Z., Liu J., Shen W., Wang Y., Wertz K., Weber P., Long J., Liu J. (2012). Enhanced autophagy plays a cardinal role in mitochondrial dysfunction in type 2 diabetic Goto-Kakizaki (GK) rats: Ameliorating effects of (-)-epigallocatechin-3-gallate. J. Nutr. Biochem..

[238] Mirza K.A., Pereira S.L., Edens N.K., Tisdale M.J. (2014). Attenuation of muscle wasting in murine C2C 12 myotubes by epigallocatechin-3-gallate. J. Cachexia Sarcopenia Muscle.

[239] Si H., Wang X., Zhang L., Parnell L.D., Admed B., LeRoith T., Ansah T.A., Zhang L., Li J., Ordovás J.M. (2019). Dietary epicatechin improves survival and delays skeletal muscle degeneration in aged mice. FASEB J..

[240] Munguia L., Ramirez-Sanchez I., Meaney E., Villarreal F., Ceballos G., Najera N. (2020). Flavonoids from dark chocolate and (-)-epicatechin ameliorate high-fat diet-induced decreases in mobility and muscle damage in aging mice. Food Biosci..

[241] Moreno-Ulloa A., Nogueira L., Rodriguez A., Barboza J., Hogan M.C., Ceballos G., Villarreal F., Ramirez-Sanchez I. (2015). Recovery of indicators of mitochondrial biogenesis, oxidative stress, and aging with (-)-epicatechin in senile mice. J. Gerontol. A Biol. Sci. Med. Sci..

[242] Hemdan D.I., Hirasaka K., Nakao R., Kohno S., Kagawa S., Abe T., Harada-Suken A., Okumura Y., Nakaya Y., Terao J. (2009). Polyphenols prevent clinorotation-induced expression of atrogenes in mouse C2C12 skeletal myotubes. J. Med. Investig..

[243] Moreno-Ulloa A., Miranda-Cervantes A., Licea-Navarro A., Mansour C., Beltra’ n-Partida E., Donis-Maturano L., De la Herra’n H.C.D., Villarreal F., A’lvarez-Delgado C. (2018). (-)-Epicatechin stimulates mitochondrial biogenesis and cell growth in C2C12 myotubes via the G-protein coupled estrogen receptor. Eur. J. Pharmacol..

[244] Mafi F., Biglari S., Ghardashi Afousi A., Gaeini A.A. (2019). Improvement in skeletal muscle strength and plasma levels of follistatin and myostatin induced by an 8-week resistance training and epicatechin supplementation in sarcopenic older adults. J. Aging Phys. Act..

[245] Kim H., Suzuki T., Saito K., Yoshida H., Kojima N., Kim M., Sudo M., Yamashiro Y., Tokimitsu I. (2013). Effects of exercise and tea catechins on muscle mass, strength and walking ability in community-dwelling elderly Japanese sarcopenic women: A randomized controlled trial. Geriatr. Gerontol. Int..

[246] Jung J., Kim Y.L., Cho H., Kim E., Jeong Y. (2019). Association between green tea consumption and sarcopenia in menopausal women: A cross sectional analysis of the Korea National Health and Nutrition Examination Survey 2008–2011. Korean J. Fam. Pract..

[247] Kim C., Hwang J.K. (2020). The 5,7-Dimethoxyflavone suppresses sarcopenia by regulating protein turnover and mitochondria biogenesis-related pathways. Nutrients.

[248] Song Y., Kim M.B., Kim C., Kim J., Hwang J.K. (2016). 5,7-Dimethoxyflavone attenuates obesity by inhibiting adipogenesis in 3T3-L1 adipocytes and high-fat diet-induced obese C57BL/6J mice. J. Med. Food.

[249] Yoshioka Y., Kubota Y., Samukawa Y., Yamashita Y., Ashida H. (2019). Glabridin inhibits dexamethasone-induced muscle atrophy. Arch. Biochem. Biophys..

[250] Lee J.W., Choe S.S., Jang H., Kim J., Jeong H.W., Jo H., Jeong K.H., Tadi S., Park M.G., Kwak T.H. (2012). AMPK activation with glabridin ameliorates adiposity and lipid dysregulation in obesity. J. Lipid Res..

[251] Hoek-van den Hil E.F., van Schothorst E.M., van der Stelt I., Swarts H.J., van Vliet M., Amolo T., Vervoort J.J., Venema D., Hollman P.C., Rietjens I.M. (2015). Direct comparison of metabolic health effects of the flavonoids quercetin, hesperetin, epicatechin, apigenin and anthocyanins in high-fat-diet-fed mice. Genes Nutr..

[252] Biesemann N., Ried J.S., Ding-Pfennigdorff D., Dietrich A., Rudolph C., Hahn S., Hennerici W., Asbrand C., Leeuw T., Strübing C. (2018). High throughput screening of mitochondrial bioenergetics in human differentiated myotubes identifies novel enhancers of muscle performance in aged mice. Sci. Rep..

[253] Yeh C.H., Shen Z.Q., Wang T.W., Kao C.H., Teng Y.C., Yeh T.K., Lu C.K., Tsai T.F. (2022). Hesperetin promotes longevity and delays aging via activation of Cisd2 in naturally aged mice. J. Biomed. Sci..

[254] Ke J.Y., Cole R.M., Hamad E.M., Hsiao Y.H., Cotton B.M., Powell K.A., Belury M.A. (2016). Citrus flavonoid, naringenin, increases locomotor activity and reduces diacylglycerol accumulation in skeletal muscle of obese ovariectomized mice. Mol. Nutr. Food Res..

[255] Ke J.Y., Kliewer K.L., Hamad E.M., Cole R.M., Powell K.A., Andridge R.R., Straka S.R., Yee L.D., Belury M.A. (2015). The flavonoid, naringenin, decreases adipose tissue mass and attenuates ovariectomy-associated metabolic disturbances in mice. Nutr. Metab..

[256] Pellegrini M., Bulzomi P., Galluzzo P., Lecis M., Leone S., Pallottini V., Marino M. (2014). Naringenin modulates skeletal muscle differentiation via estrogen receptor α and β signal pathway regulation. Genes Nutr..

[257] Burke A.C., Sutherland B.G., Telford D.E., Morrow M.R., Sawyez C.G., Edwards J.Y., Drangova M., Huff M.W. (2018). Intervention with citrus flavonoids reverses obesity and improves metabolic syndrome and atherosclerosis in obese Ldlr-/- mice. J. Lipid Res..

[258] Crespillo A., Alonso M., Vida M., Pavón F.J., Serrano A., Rivera P., Romero-Zerbo Y., Fernández-Llebrez P., Martínez A., Pérez-Valero V. (2011). Reduction of body weight, liver steatosis and expression of stearoyl-CoA desaturase 1 by the isoflavone daidzein in diet-induced obesity. Br. J. Pharmacol..

[259] Hirasaka K., Maeda T., Ikeda C., Haruna M., Kohno S., Abe T., Ochi A., Mukai R., Oarada M., Eshima-Kondo S. (2013). Isoflavones derived from soy beans prevent MuRF1-mediated muscle atrophy in C2C12 myotubes through SIRT1 activation. J. Nutr. Sci. Vitaminol..

[260] Gan M., Chen X., Chen Z., Chen L., Zhang S., Zhao Y., Niu L., Li X., Shen L., Zhu L. (2022). Genistein alleviates high-fat diet-induced obesity by inhibiting the process of gluconeogenesis in mice. Nutrients.

[261] Gan M., Ma J., Chen J., Chen L., Zhang S., Zhao Y., Niu L., Li X., Zhu L., Shen L. (2022). miR-222 Is Involved in the Amelioration Effect of Genistein on Dexamethasone-Induced Skeletal Muscle Atrophy. Nutrients.

[262] Saul D., Kling J.H., Kosinsky R.L., Hoffmann D.B., Komrakova M., Wicke M., Menger B., Sehmisch S. (2016). Effect of the lipoxygenase inhibitor baicalein on muscles in ovariectomized rats. J. Nutr. Metab..

[263] Li H., Tang S. (2021). Baicalin attenuates diet-induced obesity partially through promoting thermogenesis in adipose tissue. Obes. Res. Clin. Pract..

[264] Emanuele E., Bertona M., Pareja-Galeano H., Fiuza-Luces C., Morales J.S., Sanchis-Gomar F., Lucia A. (2016). Baicalin supplementation reduces serum biomarkers of skeletal muscle wasting and may protect against lean body mass reduction in cancer patients: Results from a pilot open-label study. Neuro Endocrinol. Lett..

[265] Kang S.I., Shin H.S., Ko H.C., Kim S.J. (2013). Effects of sinensetin on lipid metabolism in mature 3t3-L1 adipocytes. Phytother. Res..

[266] Kang S.I., Shin H.S., Kim S.J. (2015). Sinensetin enhances adipogenesis and lipolysis by increasing cyclic Adenosine Monophosphate levels in 3t3-L1 adipocytes. Biol. Pharm. Bull..

[267] Wu Y., Yang Y., Li F., Zou J., Wang Y.H., Xu M.X., Wang Y.L., Li R.X., Sun Y.T., Lu S. (2021). Icaritin Attenuates Lipid Accumulation by Increasing Energy Expenditure and Autophagy Regulated by Phosphorylating AMPK. J. Clin. Transl. Hepatol..

[268] Zhang Z.K., Li J., Liu J., Guo B., Leung A., Zhang G., Zhang B.T. (2016). Icaritin requires Phosphatidylinositol 3 kinase (PI3K)/Akt signaling to counteract skeletal muscle atrophy following mechanical unloading. Sci. Rep..

[269] Lee H., Li H., Kweon M., Choi Y., Kim M.J., Ryu J.H. (2018). Isobavachalcone from angelica keiskei inhibits adipogenesis and prevents lipid accumulation. Int. J. Mol. Sci..

[270] Hur J., Kim M., Choi S.Y., Jang Y., Ha T.Y. (2019). Isobavachalcone attenuates myotube atrophy induced by TNF-α through muscle atrophy F-box signaling and the nuclear factor erythroid 2-related factor 2 cascade. Phytother. Res..

[271] Kim D., Lee M.S., Jo K., Lee K.E., Hwang J.K. (2011). Therapeutic potential of panduratin A, LKB1-dependent AMP-activated protein kinase stimulator, with activation of PPARα/δ for the treatment of obesity. Diabetes Obes. Metab..

[272] Sa B.K., Kim C., Kim M.B., Hwang J.K. (2017). Panduratin a prevents tumor necrosis factor-alpha-induced muscle atrophy in L6 rat skeletal muscle cells. J. Med. Food.

